# Degraded neutrophil extracellular traps promote the growth of *Actinobacillus pleuropneumoniae*

**DOI:** 10.1038/s41419-019-1895-4

**Published:** 2019-09-10

**Authors:** Nicole de Buhr, Marta C. Bonilla, Jessica Pfeiffer, Silke Akhdar, Cornelia Schwennen, Barbara C. Kahl, Karl-Heinz Waldmann, Peter Valentin-Weigand, Isabel Hennig-Pauka, Maren von Köckritz-Blickwede

**Affiliations:** 1 Department of Physiological Chemistry, University of Veterinary Medicine Hannover, Hannover, Germany; 2 Research Center for Emerging Infections and Zoonoses (RIZ), University of Veterinary Medicine Hannover, Hannover, Germany; 3 Clinic for Swine, Small Ruminants, Forensic Medicine and Ambulatory Service, University of Veterinary Medicine Hannover, Hannover, Germany; 4 Institute of Medical Microbiology, University Hospital Münster, Münster, Germany; 5 Institute for Microbiology, University of Veterinary Medicine Hannover, Hannover, Germany; 6 Field Station for Epidemiology, University of Veterinary Medicine Hannover, Bakum, Germany

**Keywords:** Cell death and immune response, Immune cell death

## Abstract

*Actinobacillus pleuropneumoniae (A.pp)* causes severe pneumonia associated with enormous economic loss in pigs. Peracute diseased pigs die in <24 h with pneumonia. Neutrophils are the prominent innate immune cell in this infection that massively infiltrate the infected lung. Here we show that neutrophils release neutrophil extracellular traps (NETs) as response to *A.pp* infection. Numerous NET-markers were identified in bronchoalveolar lavage fluid (BALF) of *A.pp-*infected piglets in vivo, however, most NET fibers are degraded. Importantly, *A.pp* is able to enhance its growth rate in the presence of NETs that have been degraded by nucleases efficiently. *A.pp* itself releases no nuclease, but we identified host nucleases as sources that degrade NETs after *A.pp* infection. Furthermore, the nucleases of co-infecting pathogens like *Streptococcus suis* increase growth of *A.pp* in presence of porcine NETs. Thus, *A.pp* is not only evading the antimicrobial activity of NETs, *A.pp* is rather additionally using parts of NETs as growth factor thereby taking advantage of host nucleases as DNase1 or nucleases of co-infecting bacteria, which degrade NETs. This effect can be diminished by inhibiting the bacterial adenosine synthase indicating that degraded NETs serve as a source for NAD, which is required by *A.pp* for its growth. A similar phenotype was found for the human pathogen *Haemophilus (H.) influenzae* and its growth in the presence of human neutrophils. *H. influenzae* benefits from host nucleases in the presence of neutrophils. These data shed light on the detrimental effects of NETs during host immune response against certain bacterial species that require and/or efficiently take advantage of degraded DNA material, which has been provided by host nuclease or nucleases of other co-infecting bacteria, as growth source.

## Introduction

Peracute diseased pigs that suffer from an *Actinobacillus pleuropneumoniae (A.pp)* infection often die in <24 h. Additionally, also acute and chronic infections often occur^1^. Neutrophilic granulocytes are considered as the prominent innate immune cells in this infection^2^, but since this pathogen is producing high amount of repeats-in-toxin (RTX)-toxins that prevent phagocytosis, infiltrating neutrophils are not able to eliminate the pathogen^3^. Neutrophil extracellular traps (NETs) have been characterized as additional extracellular protective defense mechanism against various pathogens^4^. NETs are released by activated neutrophils as response to infections and mediate entrapment and partial killing by released DNA fibers decorated with histones and antimicrobial granule components^4,5^. The role of NETs during bacterial pneumonia in pigs has not been studied so far.

Shortly after the discovery of NETs, several authors demonstrated the ability of pathogenic bacteria to avoid entrapment and killing by NETs by extracellular secretion of DNA-degrading DNases^6–9^. Furthermore, some bacteria like *Streptococcus pneumoniae* use NETs for a better spreading in the body^10^.

In this study, we show that *A.pp* induces NETs but does not produce its own secreted NET-degrading nuclease. In contrast, *A.pp* is able to use NETs, which have been degraded by host nucleases or nucleases from other co-infecting bacteria as a source for NAD needed for efficient growth.

## Results

### *A.pp* induces NETs and efficiently enhances its growth in presence of degraded NETs

**Fig. 1:**
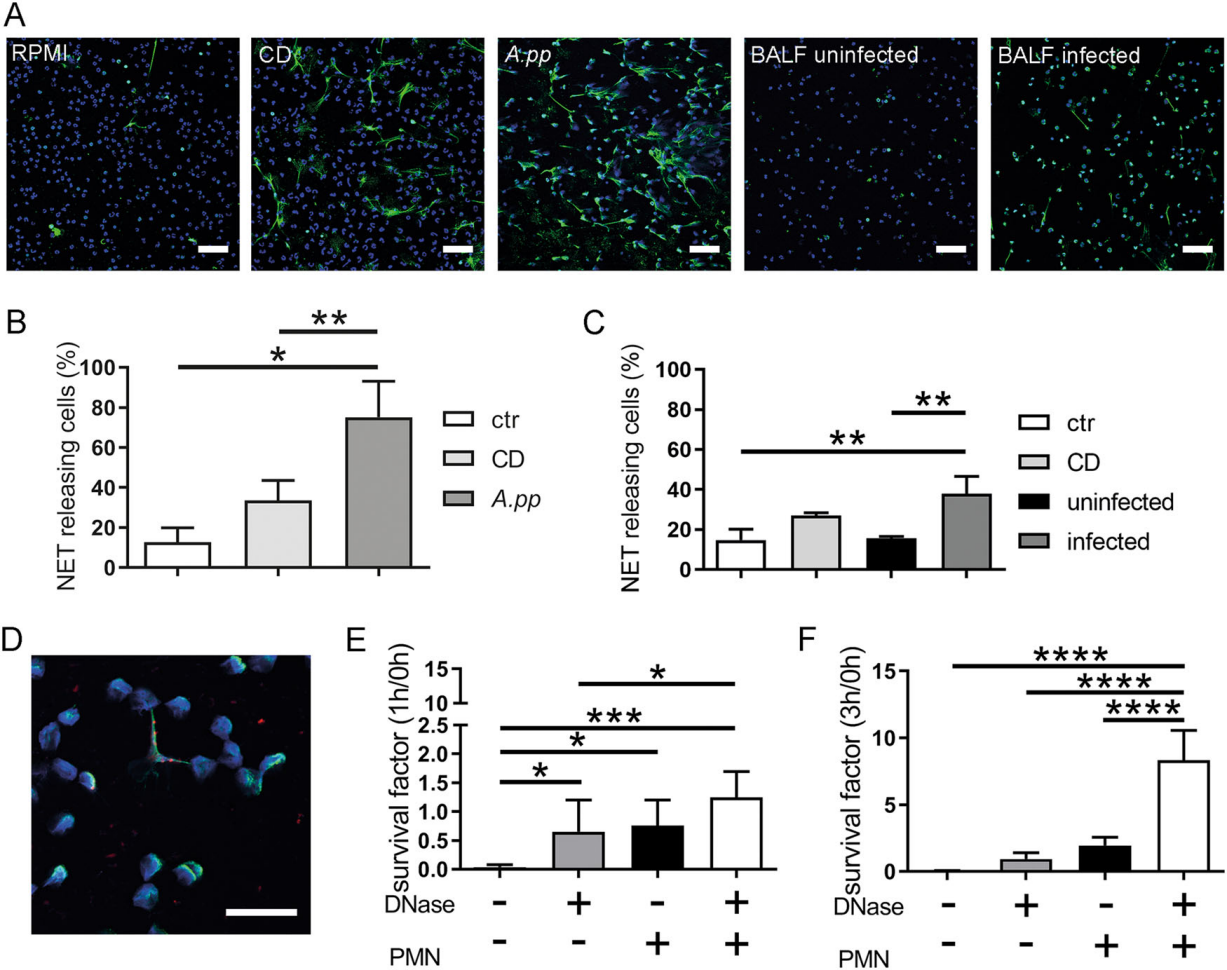
*A.pp* and BALF of *A.pp*-infected swine induce NETs, but *A.pp* grows efficiently in the presence of degraded NETs. **a** Primary fresh blood-derived porcine neutrophils were isolated and treated with RPMI as negative control (RPMI, ctr), methyl-β-cyclodextrin (CD) as positive control, *A.pp*, BALF of uninfected or *A.pp-*infected pigs. After 3 h incubation at 37 °C and 5% CO_2_ the cells were fixed. Afterwards NET staining for immunofluorescence microscopy was conducted (blue = DNA (Hoechst), green = DNA/histone‐1‐complexes (NETs)). Representative pictures are shown. (scale bar = 50 µm). **b** Statistical analysis of NET induction assays was conducted from three independent experiments. Treated cells were incubated as described in **a**. Per sample, six pictures were taken on two slides at predefined positions and the number of NET-releasing cells were determined. Compared results of the mean values are presented with ±SD (*n* = 3, one-way ANOVA *P* = 0.0083, followed by Tukey’s multiple comparison test). **c** NET induction was analyzed after treatment of neutrophils with BALF. Compared results of the mean values are presented with ±SD (*n* = 3, one-way ANOVA *P* = 0.0031, followed by Tukey’s multiple comparison test). **d** Neutrophils were incubated with *A.pp* for 3 h 37 °C, 5% CO_2_ and the cells were fixed. Afterwards staining for NETs was conducted (blue = DNA (Hoechst), green = DNA/histone‐1‐complexes (NETs), red = *A.pp*). Representative pictures are shown (scale bar = 15 µm). **e**, **f** *A.pp* was grown for 1 or 3 h at 37 °C in the absence or presence of neutrophils (PMN) and external added DNase as indicated. The survival factor was calculated based on the CFU at time point 0 h (see Fig. S2) and compared to the end point. Data shown as mean ± SD (*n* = 4, one-way ANOVA 1 h *P* = 0.0010, 3 h *P* < 0.0001, followed by Tukey’s multiple comparison test)

We investigated if NET-formation is part of the host–pathogen interaction during *A.pp*-infection. Therefore, porcine neutrophils were co-incubated with *A.pp*, or treated with sterile-filtered bronchoalveolar lavage fluid (BALF) of *A.pp-*infected or uninfected piglets. *A.pp* significantly induced after 3 h NET-formation, yet in higher amount than the positive control (Fig. 1a, b). In addition BALF of *A.pp*-infected piglets led to a significantly higher number of NET-releasing cells compared to the BALF of uninfected animals (Fig. 1c).

Since NETs are well known to entrap and inhibit the growth of bacteria, we analyzed if NETs also mediate entrapment and killing of *A.pp*. We visualized *A.pp* entrapment in porcine NETs after 1 and 3 h of co-incubation (Figs. 1d and S1), revealing that already after 1 h of co-incubation NET-structures showed partial entrapment of *A.pp*. Furthermore, intact NETs partially entrap *A.pp* after 3 h.

Second, we analyzed the survival of *A.pp* in the presence of NETs. *A.pp* was co-incubated with neutrophils for 3h and we analyzed colony forming units (CFU/ml) (Fig. S2) and calculated the survival factor (SF) of *A.pp* (Figs. 1e, f and S2). Interestingly, a significantly higher survival (SF_3h_ = 2.08) was determined in the presence of neutrophils compared to growth controls in the absence of neutrophils. Furthermore, a highly significant increase in growth of *A.pp* was detected, when NETs were degraded by external added DNase (SF_3h_ = 8.85), whereas DNase alone (in the absence of neutrophils or NETs) led to a significantly lower survival (SF_3h_ = 1.01).

**Fig. 2:**
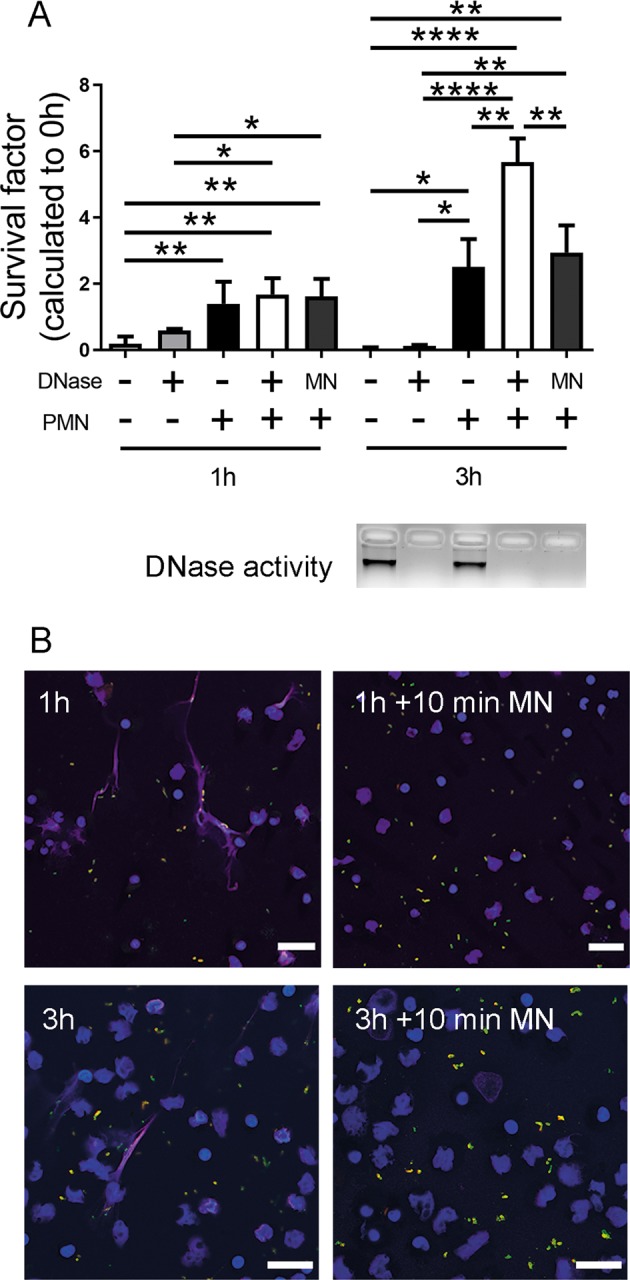
Control experiments to quantify the amount of bacteria sticking to NETs and to evaluate phagocytic activity of neutrophils. **a** *A.pp* was grown and the survival factor was calculated as described in Fig. 1e and f. The sample with neutrophils and *A.pp* was treated with micrococcal nuclease (MN) for 10 min at the end of incubation to release bacteria entrapped in NETs. The efficacy of treatment was verified with DNase activity test (agarose gel electrophoresis), as well as immunofluorescence microscopy (right panel in **b**). Data are shown as mean ± SD (*n* = 3, one-way ANOVA 1 h *P* = 0.0010 and 3 h *P* < 0.0001, followed by Tukey’s multiple comparison test). **b** Neutrophils were incubated with *A.pp* for 3 h 37 °C, 5% CO_2_ and the cells were fixed. Afterwards staining for NETs and *A.pp* was conducted (blue = DNA (Hoechst), magenta = DNA/histone‐1‐complexes (NETs), red and green = extracellular *A.pp*, green = intracellular *A.pp*). Representative pictures are shown (scale bar = 20 µm). For the pictures the main focus was to present neutrophils and NET structures. Therefore, the pictures are zoom pictures on specific areas and adjusted to the focus level for neutrophils/NETs. Amount of A.pp visible in different focus planes of images varies a lot, thus, the shown image does not necessarily reflect average A.pp level found per sample

The SF is based on accurately knowing the total amount of bacteria at the start and end time point. However, if bacteria are entrapped by NETs, we needed to exclude that the increased SF in the presence of degraded NETs is not only a simple phenomenon of releasing entrapped bacteria from NETs instead of a growth-promoting effect. As control experiment, we repeated the survival assay and included a control sample, where we added micrococcal nuclease (MN) to the sample *A.pp* + PMN at the end of the experiment for only 10 min to additionally release bacteria that are possibly simply sticking to intact NETs (Fig. 2a). The efficiency of the short-time (10 min) MN treatment to degrade NETs was confirmed in Figs. 2b and S3. As shown in Fig. 2a, the determination of SF revealed no significant difference compared to the sample without nuclease treatment indicating that the lower recovery rate in those samples is not just because of bacteria sticking to the NETs.

**Fig. 3:**
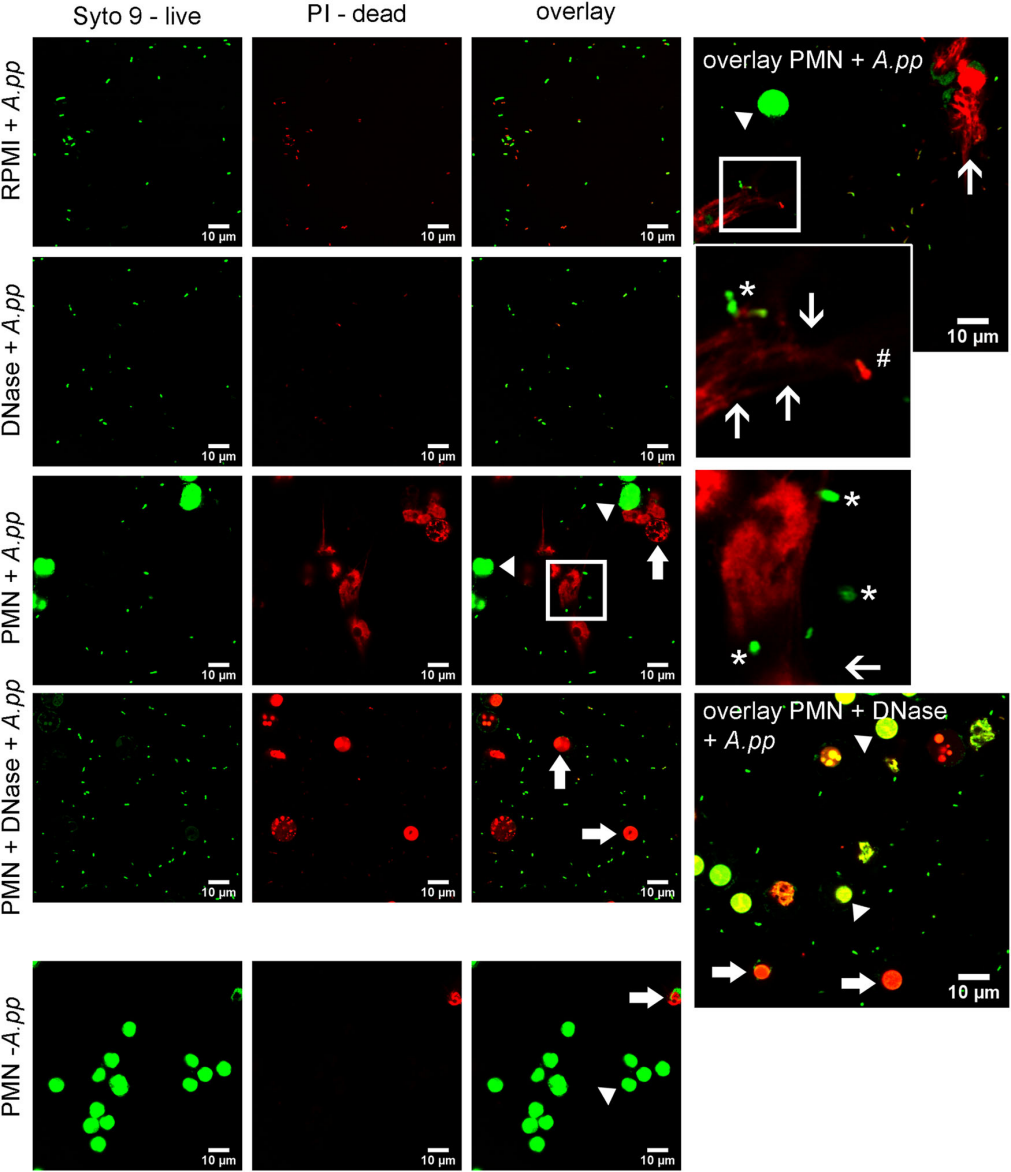
Baclight assay to determine dead versus viable bacteria in the presence of neutrophils. Live/dead bacterial viability staining of *A.pp* in the presence of absence of neutrophils was conducted (green = living cell/*A.pp*, red = dead cell/*A.pp*). Samples were incubated for 1 and 3 h as described in Fig. 1f. Per sample 8–12 pictures were taken and representative pictures from one experiment, time-point 3 h are shown (scale bar = 10 µm). Arrow head = living neutrophil, arrow = dead neutrophil, open arrow = NET structures, white *living *A.pp*, white #dead *A.pp*. As control for the neutrophils an untreated sample was included (PMN—*A.pp*)

In addition, in Fig. 3 we present images of live/dead bacterial viability staining to determine viability of *A.pp* in the presence of neutrophils or entrapped in NETs after 3 h of co-incubation: Viable bacteria are shown in green (white star) and dead bacteria in red (white #). In good correlation to the data shown in Figs. S2 and 1f, there is no bacterial growth, and some bacteria are dying in the absence of neutrophils and DNase (RPMI control). In contrast, in all other samples, especially in the presence of neutrophils, high numbers of viable bacteria are detectable. Bacteria entrapped in NETs are also only partially killed (red), as viable (green) bacteria are found in NETs.

Furthermore, no hints for high phagocytosis rate were found by an intra-/extracellular staining of bacteria (Fig. 2b, S3). This goes in line with previous literature, indicating that *A.pp* is producing high amount of RTX-toxins that prevent phagocytosis^3^. Summarizing we conclude that neutrophils are not able to efficiently kill *A.pp* by phagocytosis or entrapment within NETs.

However, our data so far lead to the suggestion that *A.pp* is able to induce NETs and uses degraded NETs as growth factor. As a next step, we aimed to investigate if this phenomenon is relevant in vivo.

### NETs are formed and degraded during *A.pp* infections in vivo

**Fig. 4:**
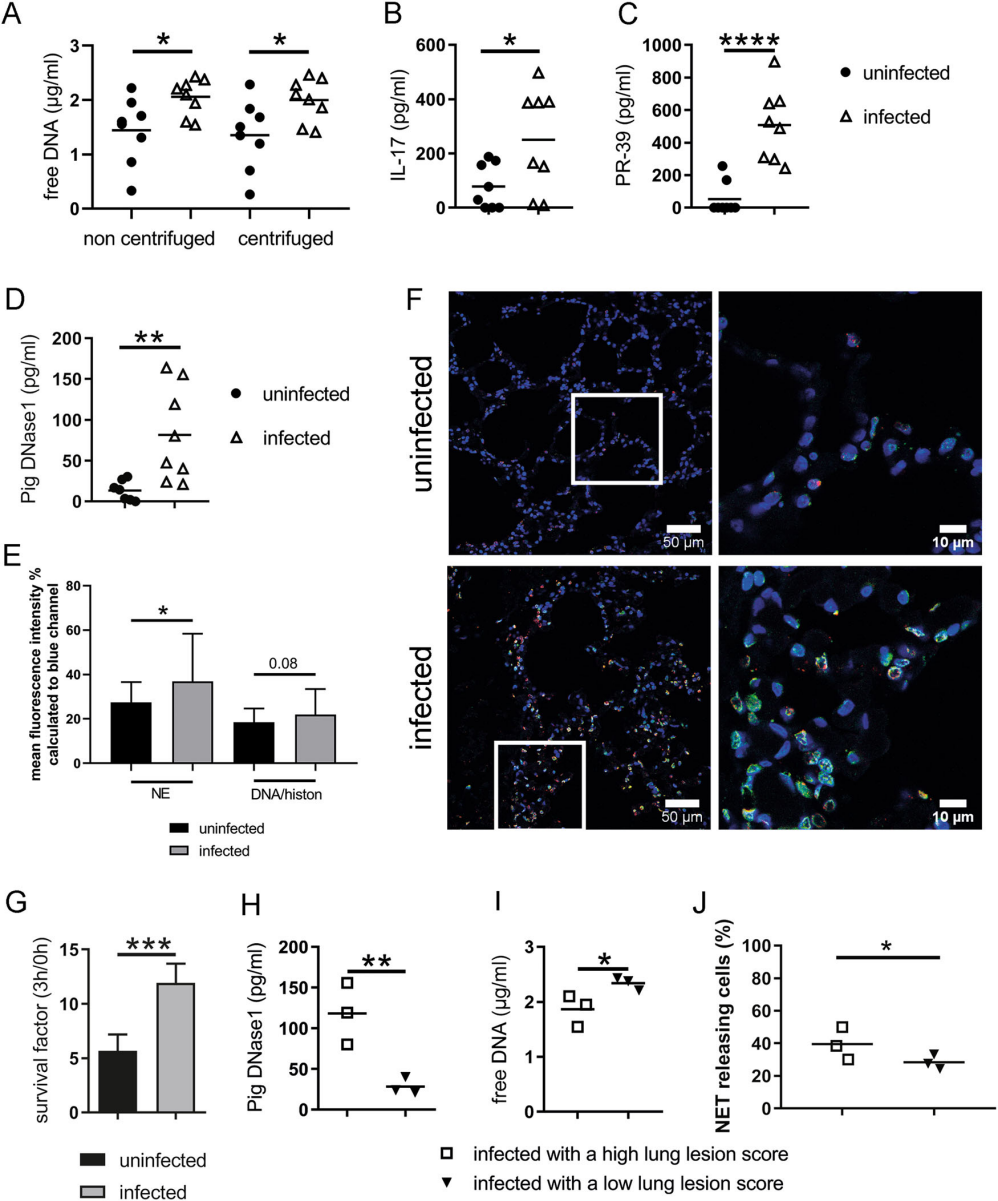
Signs for NETs found in *A.pp*-infected swine and correlate with the severity of infection. BALF samples (*n* = 8 for each group) were analyzed for NET markers **a**–**c** and DNase1 **d**. **a** The amount of free DNA in BALF samples was determined using Pico Green assay. Data of four independent technical replicates are presented as mean. **b**–**d** Samples were analyzed by ELISA. The mean of duplicates for each animal was calculated. **e**, **f** Stained lung slices of three animals per group were analyzed by ImageJ software to determine the percentage of mean fluorescence of red (NE) and green (DNA/Histone) signal based to the blue signal (nuclei). Per animal 10 immunofluorescence microscopy pictures were analyzed (uninfected *n* = 29, infected *n* = 30). High amount of neutrophil elastase and histone signals were detectable in lungs of *A.pp*-infected swine. Representative pictures of immunofluorescence microscopy are shown (blue = DNA (Hoechst), green = DNA/histone‐1‐complexes (NETs), red = neutrophil elastase). The right picture is a magnification from left (scale bar left pictures = 100 µm, scale bar right picture = 25 µm). **e**, **g** *A.pp* was grown 3 h at 37 °C in the presence of filtered BALF samples. To exclude that PR-39 as antimicrobial peptide influences the survival of bacteria, in the uninfected group three BALF samples without PR-39 detection (0 pg/ml) were used. The mean of DNase I in these samples are 10.82 pg/ml. In the infected group three BALF samples of pigs with the highest amount of PR-39 were used (640–897 pg/ml). The mean of DNase I in these samples are 97.17 pg/ml. The survival factor was calculated based on the CFU at start point compared to 3 h. The experiment was conducted in three independent technical replicates with all six tested samples each run. **h**–**j** In addition, the results of free DNA and pig DNase1, as well as NET releasing cells after BALF treatment for 3 h (from Fig. 1c) are grouped in high and low LLS (three pigs each group from the infected group). Information of statistical analysis: Compared results are shown as mean ± SD in all graphs. In **g**, **j** data are analyzed with one-tailed paired Student’s *t*-test. In all other graphs data are analyzed with one-tailed unpaired Student’s *t*-test (*P < 0.05, **P < 0.01, ***P < 0.001, ****P < 0.0001)

To determine the presence of NETs during the early phase of an *A.pp* infection in the lung of experimentally infected piglets, we analyzed and compared BALF of *A.pp*-infected versus uninfected pigs for NET-specific markers. Free DNA is described as the most prominent marker for NETs^11^. First, we measured a significantly higher amount of free DNA in BALF of infected piglets (Fig. 4a) compared to uninfected piglets. As free DNA is not exclusively a NET marker, we analyzed the BALF for IL-17 and the antimicrobial peptide (AMP) PR-39. These specific markers were used since a massive IL-17 release can be detected during NET-formation and PR-39 is stored in neutrophil granules and can be found in porcine NETs. Thus, both parameters are further indicators for NET-formation^5,12^. Furthermore, it is hypothesized, that neutrophil recruitment is due to IL-17 and triggers progressive inflammation during *A.pp* infections^13^. Importantly, ELISA-based quantification showed significantly increased amounts of IL-17 and PR-39 in BALF of infected pigs compared to uninfected pigs (Fig. 4b, c).

Next, we analyzed histological lung tissue slices by immunofluorescence microscopy. Indeed, in infected piglets high number of cells positive for histone–DNA 1 complexes and neutrophil elastase as marker for NETs were detectable compared to uninfected piglets (Fig. 4e, f). However, no clear NET-fibers were visible (Fig. 4f) indicating that NETs are degraded. The host releases nucleases, as DNase1 with the function to clear NETs and to avoid detrimental effects of NETs^14^. In good correlation, significantly more pig DNase1 (Fig. 4d) as most prominent nuclease was detected in BALF of infected pigs compared to uninfected pigs, but not Pig DNase2 (Fig. S4). Furthermore, in accordance with our hypothesis that degradation of NETs in the presence of nucleases may contribute to *A.pp* growth, *A.pp* showed higher SF in BALF of infected animals with higher host DNase1 activity compared to BALF of uninfected animals (Fig. 4g, mean uninfected = 10.82 pg/ml, mean infected = 97.17 pg/ml).

Next, we correlated the amount of host DNase1 and free DNA in BALF of infected pigs with high and low pathological lungs lesion score (Fig. 4h, i, Table S1). In the BALF of piglets with high lung lesion score (LLS) lower amounts of free DNA but higher values of pig DNase1 was detected compared with piglets having low LLS. In good correlation to these data, bioanalyzer-based analysis of DNA fragments inside BALF and samples from experiments from Fig. 2a showed differences in the DNA fragment size (Fig. S5): BALF from pigs with high LLSe, high amount of DNase1 and low amount of free DNA showed more low-molecular DNA fragments in bioanalyzer analysis and less high-molecular DNA in agarose gel electrophoresis analysis. On the other hand, BALF from pigs with low LLSe, lower amount of DNase1 and higher amount of free DNA showed less low-molecular DNA fragments in bioanalyzer analysis and more high-molecular DNA in agarose gel electrophoresis analysis (Fig. S5).

Finally, BALF of infected pigs with high LLS induced significantly more NETs compared to BALF of pigs with low LLS (Fig. 4j). In summary, these data confirm that (1) NETs are formed and again degraded in vivo as response to *A.pp* infection and (2) underline the hypothesis that degraded NETs may promote the growth of *A.pp* in the lung and thereby contribute to severe lung damage.

### *A.pp* does not produce its own NET-degrading nucleases, but is hijacking other nuclease sources for its growth benefit in the presence of NETs

We were interested if *A.pp* produces extracellular DNase(s) to degrade NETs as a growth benefit. However, testing supernatants of *A.pp* and fresh grown *A.pp* did not show any activity of extracellular nucleases in case of *A.pp* serotype 2 using different functional in vitro assays (Fig. S6A, B). A DNase-activity test with BALF samples demonstrated a DNase activity independent if the BALF was analyzed from uninfected or infected piglets (Fig. S6C), confirming the availability of host-derived nucleases as shown above. Furthermore, BLAST-analysis of several known NET-degrading secreted nucleases of bacteria in the genome sequence of *A.pp* did not give a hint for the presence of secreted nucleases in the *A.pp* serotype 2 genome (Table S2).

**Fig. 5:**
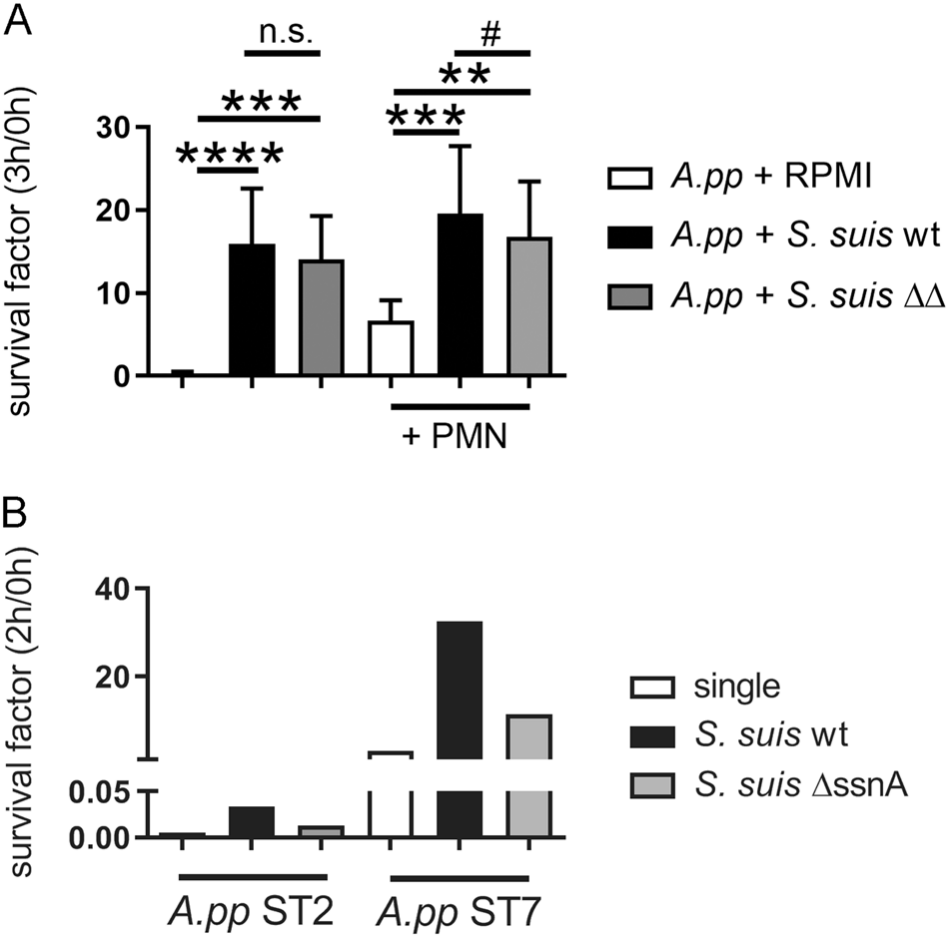
Degraded NETs of co-infecting bacteria as a source for growth factors promote growth of *A.pp*. **a** *S. suis* was used in a co-infection assay as a DNase source in the absence or presence of porcine neutrophils. Furthermore, the DNase-deficient mutant *S. suis*Δ*ssnA* Δ*endAsuis* was used as control. The survival factor was calculated based on the CFU at 0 and 3 h. Data shown are mean ± SD (*n* = 6, one-way ANOVA −PMN *P* *=* 0.0006, +PMN *P* *<* 0.0001, followed by Dunnet’s multiple comparison test to RPMI and ^#^*P* *<* 0.01, one-tailed paired Student’s *t*-test). **b** Whole blood of four piglets from the same sow were incubated with *A.pp* serotype 2 or 7 as single infection or co-infection with *S. suis* wildtype (wt) or the respective DNase mutant (Δ*ssnA*) for 2 h. The CFU/ml was determined by plating and the survival factor (SF) calculated. In blood of three piglets *A.pp* was efficiently killed with a SF < 0.05. Data presented from the survival of *A.pp* in the blood of one piglet

One of the major co-infection microorganisms in the upper respiratory tract of pigs is *Streptococcus* (*S.*) *suis*. This pathogen has been shown by us in former studies to produce two DNases (EndAsuis and SsnA) that can both degrade NETs, but SsnA being the most efficient NET evasion factor^6^. A nuclease-deficient double mutant of *S. suis* exhibited significant lower growth benefit for *A.pp* compared to its respective nuclease producing wildtype strain (Fig. 5a). Thus, we speculate that bacterial DNases derived from co-infecting or co-colonizing bacteria may contribute, besides host DNases, to enhance the growth of *A.pp* during pneumonia.

In Table S3, we show data about the prevalence of lung co-infections with *S. suis* and *A.pp* in pigs with respiratory disorders. In 559 pigs, ~11% showed co-infection of *A.pp* and *S. suis* in the lung. Furthermore, a severe clinical case of pleuropneumonia in gilts originating from a vaccinated *A.pp*-free breeding farm illustrates the impact of *A.pp* and *S. suis* co-infection on the health status of herds (Table S4). In line with this hypothesis, we detected SsnA as the major nuclease contributing to NET evasion^15^ by dot-blot analysis in several BALF of pigs from the presented experimentally infected piglets as well as field samples (Fig. S7). This underlines our hypothesis that secreted DNases from the host, as well as co-infections are present in the lung and may contribute to growth of *A.pp*.

Finally, we were interested if a co-infection with a nuclease-producing *S. suis* strain triggers *A.pp* survival ex vivo in whole blood. The survival of *A.pp* serotype 2 and 7 was determined in whole blood in presence of *S. suis* compared to a nuclease-deficient SsnA mutant. Since most animals have high antibody titer against *A.pp*, in most individuals blood is efficiently able to eliminate *A.pp*. For this reason, the assay was conducted with the blood of four piglets from the same age and sow to screen for animals that do not clear *A.pp* from blood. In the blood of three piglets *A.pp* was efficiently eliminated as expected. However, in the blood of one piglet, we confirmed the phenomenon as seen in Fig. 5a: The nuclease-producing wildtype strain shows a higher growth benefit for *A.pp* compared to the respective SsnA mutant strain (Fig. 5b). Thus, it may be hypothesized that *S. suis* nucleases in some individuals that are susceptible to *A.pp* survival in blood additionally trigger growth of *A.pp* by degrading NETs and thereby contribute to severity of lung diseases caused by *A.pp* as described in the case report.

### *A.pp* benefits from adenosine and NAD, which is freely available in degraded NETs and dead bacteria

We aimed to understand why degraded NETs promote the growth of *A.pp*. Growth of pathogenic *A.pp* depends on an external NAD source^16^. Therefore, we tested if degraded NETs act as external NAD source.

**Fig. 6:**
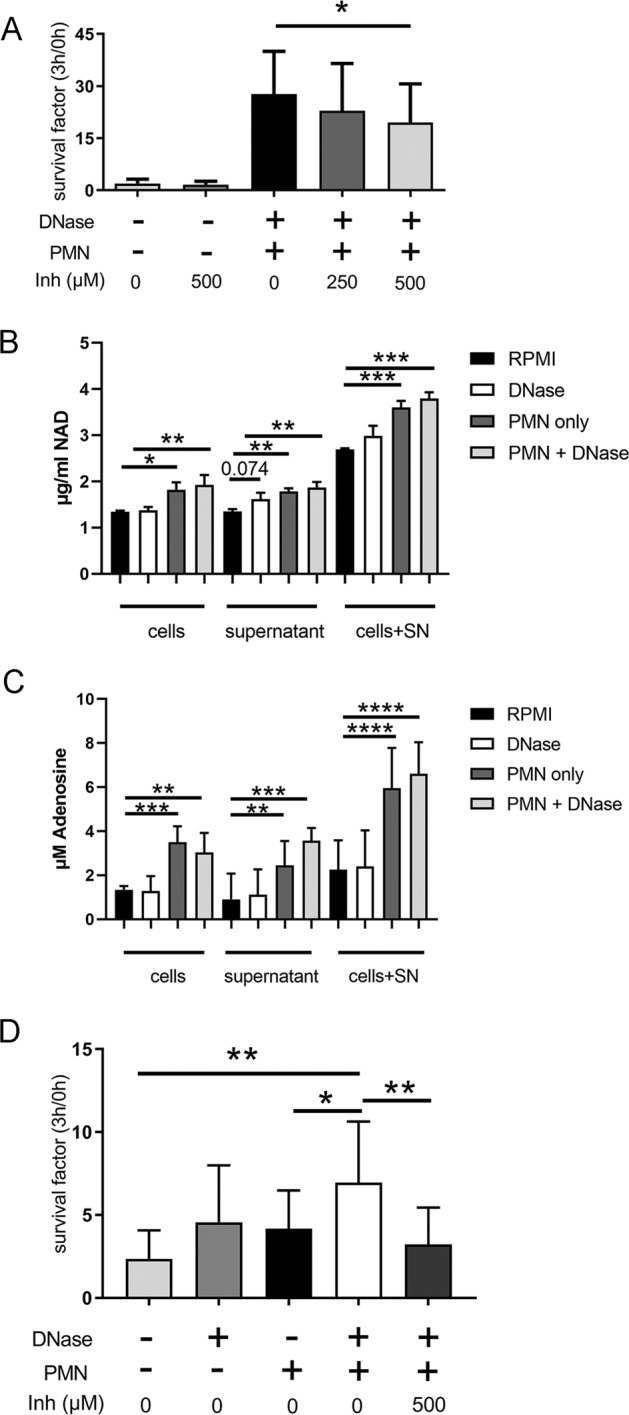
NAD and adenosine as growth factor for *A.pp* and released from NETs. **a** *A.pp* was grown for 3 h at 37 °C in the absence or presence of neutrophils (PMN) and DNase as described in Fig. 1e and f. A 5′-nucleotidase inhibitor was used to reduce adenosine production. The survival factor was calculated. Data shown are mean ± SD (*n* = 4, samples with and without inhibitor were analyzed with one-tailed paired Student’s *t*-test). **b**, **c** NAD was determined with ELISA and adenosine with a colorimetric assay in samples from NETs antimicrobial activity assay (Fig. 1e, f) but without *A.pp*. Data presented from *n* = 3 independent experiments. Data are presented with mean ± SD (*n* = 3, measured in one technical detection run, one-way ANOVA in each sample group, followed by Dunnett’s multiple comparison test to RPMI). **d** *H. influenzae* was grown for 3 h at 37 °C in the absence or presence of neutrophils (PMN) and DNase as described in Fig. 1 for *A.pp*. A 5′-nucleotidase inhibitor was used to reduce adenosine production. The survival factor was calculated and revealed that the inhibitor suppressed the growth benefit in the presence of DNase or degraded NETs. Data shown are mean ± SD (*n* = 3, ANOVA *P* = 0.0017, followed by Tukey’s multiple comparison test)

We repeated the assay from Fig. 1f by adding an adenosine synthase inhibitor to block the production of adenosine and consequently the NAD production by *A.pp* from available components. Interestingly, when treating *A.pp* with this inhibitor, the growth of *A.pp* in the presence of degraded NETs was significantly, but not completely, diminished (Fig. 6a), suggesting that NETs may serve as an external adenosine source that promotes growth of *A.pp*. Control experiments in the absence of NETs/neutrophils confirmed a complete growth inhibition of *A.pp* in presence of the inhibitor (Fig. S8).

We further analyzed this samples (Fig. 1f) for free available NAD and adenosine, which is significantly available higher in samples containing neutrophils. The highest values were measured in samples with neutrophils and externally added DNase (Fig. 6b, c). Data received in the presence of *A.pp* as consumer revealed distinctly less available NAD and adenosine, and confirmed usage of adenosine and NAD by *A.pp* (Fig. S9). These data underline our hypothesis that neutrophils and degraded NETs may serve as a source for growth-promoting NAD.

To confirm this phenomenon in vivo, we measured NAD in BALF samples of uninfected and infected piglets and detected comparable amounts in both groups (Fig. S9C). Again a comparison of infected pigs with high versus low LLS was conducted and revealed that piglets with high LLS showed remarkably higher NAD values in BALF compared to piglets with low LLS (Fig. S9D). This is quite important that the difference is visible despite usage of NAD by the bacteria present in the lung and, thus, correlate well with our hypothesis that degraded NETs may be a source for *A.pp* growth and contribute to a more severe phenotype of disease.

Finally, we aimed to see if other related bacteria, which also require NAD for its growth show a similar phenotype. *Haemophilus* (*H.*) *influenzae* colonizes the upper respiratory tract of humans, but can also lead to severe chronic lower respiratory tract diseases^17^. Indeed we found a similar phenotype as for *A.pp* after incubation of *H. influenzae* in the presence of human neutrophils (Fig. 6d). Degraded NETs significantly promote the growth of *H. influenzae*. In good accordance to our hypothesis that degraded NET are used as source for NAD, the 5′-nucleotidase inhibitor significantly suppressed the growth benefit in the presence of degraded NETs.

## Discussion

Our results indicate that NET-formation is part of the host–pathogen interaction during *A.pp* infections in pigs (Fig. 4)*. A.pp* itself induces high amounts of NETs compared to other porcine pathogens like *S. suis*^6^ and is partially entrapped inside NETs (Figs. 1 and S1).

Also during ex vivo experiments, BALF of *A.pp-*infected pigs lead to a significantly higher amount of NET-releasing cells (Fig. 1c). Thus, it can be hypothesized that during an in vivo infection various different factors in the lungs may stimulate the infiltrating neutrophils to produce NETs. LPS has been demonstrated to induce indirectly via activated platelets NETs^18^. However, LPS was detected in BALF of all pigs (Fig. S10) and cannot be the key NET-inducing factor in BALF of infected animals. Further NET-inducing factors could be bacterial and/or host-derived factors. It is conceivable that PR-39 like the human AMP LL-37 triggers NET-formation in the porcine lung^19^, as this AMP is detected in significantly higher amount in BALF of *A.pp-*infected pigs (Fig. 4c). However, to determine the processes that lead to NET-formation during *A.pp* infection in the lung, further experiments are needed in the future.

Independent of the NET-inducing factor it was surprising that *A.pp* efficiently increases its growth in the presence of degraded NETs (Figs. 1 and S2). Therefore, we hypothesized that *A.pp* is not only resisting the antimicrobial activity of NETs, *A.pp* is rather able to use degraded NETs for its own growth benefit. The most obvious explanation would be the production of a bacterial DNase as shown as NET-evading factor produced by several other bacteria^7–10,15^. Our results indicate that *A.pp* does not produce its own secreted NET-degrading DNase (Figs. 1e, f, S1, S6A–C, Table S2). Nevertheless, the survival of *A.pp* is more efficient in the presence of degraded NETs by adding an external DNase (Fig. 1e, f). Additionally, it has to be mentioned that DNase alone also leads to a slight but detectable growth benefit for *A.pp*. This phenomenon might be explained by dying *A.pp* that also provide small amount of DNA, which can be used by growing *A.pp* as growth source similar to degraded NETs (Figs. 1e and 3).

We identified different possible candidates as DNases source in vivo. The host-derived pig DNase1 was detected with significantly higher amount in the infected versus uninfected group. Interestingly, as Fig. S6C demonstrates DNase activity of BALF samples, detection of pig DNase1 was not in all cases associated with DNA degradation. Therefore, possible other DNases of the host as well as other bacterial DNases could be a source for nuclease activity. Lactoferrin, a typical lung disease marker in humans and animals^20^, was considered to exhibit nuclease activity^21^. ELISA-based analysis detected lactoferrin in BALF, but no significant difference was found comparing BALF from infected versus uninfected animals (Fig. S6D). Similarly, we detected in all BALF samples pig DNase2 (Fig. S4).

Next, to host-derived DNases, we analyzed in addition if DNases of a co-infection could influence the *A.pp-*infection. One major co-infection microorganism in the upper respiratory tract of pigs is *S. suis*. The nuclease SsnA of *S. suis* is described to be effective active in a neutral pH^6^ and was also confirmed to trigger *A.pp* growth during in vitro co-infection experiments in the presence of neutrophils (Fig. 5a, b) or also in the absence of neutrophils (Fig. S11). Based on this finding, we hypothesize that bacterial DNases derived from co-infection may contribute, besides host DNases, to enhance the growth of *A.pp* during pneumonia. This is an important finding when considering that co-infections of *A.pp* with, e.g. *S. suis* are suspected to lead to severe clinical cases despite vaccination as presented in a recent clinical case (Table S4). In addition, the prevalence of lung co-infections with *S. suis* and *A.pp* in respiratory disorders takes places in the same lung compartments (Table S3). Independent of the infection status we could furthermore detect SsnA in several BALF of pigs (Fig. S7). We conclude that neutrophils and NETs which are produced in response to *A.pp* stimulation in high amounts (Fig. 1) and degraded by host or bacterial nucleases do promote the growth of *A.pp*. Considering potential future therapeutic interventions, we were wondering about the biochemical explanation of this phenomenon.

Since it is well known that growth of pathogenic *A.pp* depends on external NAD sources^16^, we speculated if degraded NETs may act as external NAD source. Control experiments confirmed that *A.pp* uses NAD or DNA as external source to promote its growth and that this effect can be diminished by inhibiting the 5′-nucleotidase (Fig. S8). A BLAST showed a highly similar enzyme of the 5′-nucleotidase in *A.pp*, which is in addition described in *H. influenzae*. The 5′-nucleotidase is described as an essential enzyme in the NAD and adenosine metabolism of cells and bacteria^22^.

Recent findings showed that NETs can be converted to deoxyadenosine by pathogenic bacteria and thereby promote immune cell death^23^. The generation of deoxyadenosine from NETs requires two enzymes, nuclease and adenosine synthase (with 5′-nucleotidase signature sequence)^23^. Adenosine 5′-(α,β-methylene) diphosphate is known as a 5′-nucleotidase inhibitor that reduces adenosine production by blocking adenosine synthesis^24^. The addition of this inhibitor diminished the growth benefit of *A.pp* in the presence of degraded NETs (Fig. 6a). In addition, we confirmed here that NAD and adenosine are freely detectable in the presence of neutrophils and degraded NETs (Fig. 6b, c). We hypothesize that *A.pp* is able to use these substrates to promote its own growth (Figs. 6 and S9). Since this phenomenon is partially also visible in the absence of neutrophils, we assume that in general DNA, which also appears from dying bacteria, e.g. by killing bacteria with PR-39 leads to a similar phenomenon. PR-39 is bound to *A.pp*-induced NETs (Fig. S12A). Antimicrobial activity of PR-39 can be diminished in the presence of external DNase (Fig. S12B, C). This phenomenon can be explained by a usage of DNA from PR-39-killed *A.pp* by growing *A.pp*. The external DNase degrades DNA and 5′ nucleotidase of *A.pp* is active as described. In general, DNA fragments contain high amount of NAD parts, namely adenosine, this could be one factor facilitating the survival of *A.pp* in the presence of neutrophils or dying bacteria if the 5′-nucleotidase is active. The synthesis of NAD can chemically be initiated from adenosine monophosphate (AMP)^25^. Interestingly, nicotinamide mononucleotide (NMN) was described as the key intermediate, which is part in the NAD^+^ utilization pathway in *Pasteurellaceae* like *A.pp* and can serve as a substrate for production of NAD^26^. Therefore, it is conceivable that *A.pp* uses DNA as source for NAD production.

Interestingly, NAD and adenosine are also described as a signaling molecule for immune cells, for example adenosine act as chemotactic stimulus for neutrophils via purinergic receptor A1R^27^. In addition purines like deoxyadenosine produced by *S. aureus* lead to apoptosis of immune cells and adenosine is described for *S. aureus* as an escape molecule from NETs^23,24,28^. Therefore, our findings complete the complex knowledge about the function of NAD and adenosine in the host–pathogen interaction with new insights on neutrophils and NAD-dependent bacteria like *A.pp*.

Looking to other bacteria closely related to *A.pp*, like the human pathogen *H. influenzae*, one conceivable scenario is that this pathogen benefits from a similar mechanism. *H. influenzae* colonizes the upper respiratory tract of humans, but can also lead to severe chronic respiratory tract diseases^17^. Importantly, nontypeable *H. influenzae* is described to induce NETs in human neutrophils^29^. In this context it is highly interesting that a recent publication including clinical data showed that NET-formation is increased in patients with severe COPD and associated with a loss of microbiota diversity including dominance of *Haemophilus* species^30^. *H. influenzae* also needs external NAD. Furthermore, in the respiratory tract of humans several bacteria are known that produce NET-degrading DNases like *S. pneumoniae*, *S. mitis*, and *Staph. aureus*^9,10,31^. Indeed we found a similar phenotype as for *A.pp* after incubation of *H. influenzae* in the presence of human neutrophils (Fig. 6d). Thus, future work should be performed to study similar effects in other infectious diseases, especially during co-infections to see if degraded NETs might promote growth of pathogens that require NAD. This might be of high clinical importance, since nucleases are considered as therapeutical strategy for severe chronic lung infections^32,33^.

Here in our study a concentration of 100 U/ml (~0.08 mg/ml) nuclease activity is used to show the effect that degraded NETs promote growth of *A.pp* and *H. influenzae*. In comparison, the following values can be found in the literature for therapeutic values for nuclease activity: When using nuclease for treatment of CF patients, concentrations of 2.5 mg per lung are applied^34^ and a concentration of 2 µg/ml DNase1has been shown to reduce pathogenic effects of cigarette smoke exposure in the lung^35^.

Based on our presented work, it might be taken into account that a therapeutical nuclease treatment during chronic lung diseases which degrades present NETs^32^ might highly impact the growth of certain NAD-dependent pathogenic bacteria that can use degraded NETs as growth factor.

In conclusion, our study presents for the first time evidence that a pathogen induces NET-formation to take advantage of resulting DNA fragments and free available NAD and adenosine for its own growth, thus exploiting external sources of DNases, e.g. host nucleases or nucleases derived from co-infecting bacteria. This is a new mechanism how bacteria may benefit from NETs and host nucleases or nucleases of co-infecting pathogens. These findings may help to develop new therapeutic strategies, especially in case of co-infection and high amount of NETs. Interestingly, viruses, e.g. influenza have been shown to induce NETs in lungs and thereby trigger co-infection with, e.g. *S. pneumoniae* and lead to severe pneumonia or otitis media^36^. As we have shown that *A.pp* and *H. influenzae* benefit from free available DNA-fragments, adenosine, and NAD of degraded NETs, it may also be hypothesized that an acute virus infection induce NETs and subsequently provide nutrients for sleeping *A.pp* or *H. influenzae* as starting point of a fatal lung infection. In piglets infections with viruses like the porcine reproductive and respiratory syndrome virus are also a major cause of lung infections and can increase the susceptibility to bacterial infections^37^. However, more studies are needed to prove this hypothesis.

## Methods and materials

### Ethics statement

The in vivo infection experiment with piglets had been approved by the Ethics and Animal Welfare Committee of the University of Veterinary Medicine Hannover and the local permitting authorities in the Lower Saxony State Office for Consumer Protection and Food Safety (approval number: 33.9-445 42502-12/0835).

The collection of fresh heparinized blood from healthy pigs was approved by Lower Saxony State Office for Consumer Protection and Food Safety (Niedersächsisches Landesamt für Verbraucherschutz und Lebensmittelsicherheit) under nos. 12A243 and 18A302.

The collection of fresh heparinized blood from healthy human donors was approved by the ethic committee of the Hannover Medical School (MHH), Hannover, Germany and registered under no. 3295-2016.

### *A.pp* growth conditions

*A.pp* serotype 2 strain C3656/0271/11 was mainly used in this study. This strain was isolated during routine diagnostics at the Institute of Microbiology, University of Veterinary Medicine Hannover, Germany from the lung tissue of a diseased fattening pig during an *A.pp* outbreak^38^.

*A.pp* serotype 7 strain AP76 was only used in the whole blood killing assay in this study. This strain was isolated during routine diagnostics in the Western College of Veterinary Medicine, Saskatoon, Canada from the lung of a diseased pig^39,40^.

*A.pp* was grown on boiled-blood agar plates with nicotinamidadenindinucleotide (NAD) at 37 °C and 5% CO_2_ (weekly prepared from −80 °C cryo-stock). Liquid culture was grown in fresh pleuropneumonia-like organism medium (PPLO) with 0.1% Tween 80 and 12 ml BBL IsoVitaleX (BD) per litre PPLO medium (named as PPLO).

For in vivo experiments *A.pp* was grown in PPLO medium to an OD_600nm_ = 0.45 during an overnight culture. This culture was diluted 1:1000 in 154 mM sterile sodium chloride solution.

For in vitro experiments 10 ml PPLO medium was inoculated with one colony-forming unit from the boiled-blood agar plate and incubated for 16 h in a culture tube (12 ml culture tube, Simport) placed in a box filled with slowly melting crushed ice at 37 °C and 5% CO_2_. On the next day 5 ml over-night-culture were transferred to 45 ml of prewarmed PPLO medium and afterwards incubated in a rotation shaker (200 rpm, 37 °C) until reaching the OD_600nm_ = 0.6 (mid-log-phase). Afterwards the bacterial suspension was freshly used for in vitro experiments. Therefore, 2 ml bacterial suspension was centrifuged (2600 × *g*, 5 min) and the pellet of bacteria was washed once with 1× PBS. The bacterial suspension was adjusted to an OD_600nm_ = 0.6 in 1× PBS. Due to variations occurring based on usage of freshly grown bacteria, a MOI of 0.1–2 was reached for all assays.

### Purification of porcine and human neutrophils

Porcine neutrophils were purified using Biocoll (1.077 g/ml; Merck Millipore) and hypotonic lysis of erythrocytes as previously described^41^. Human neutrophils were isolated from fresh blood of healthy donors using the PolymorphPrep system (Axis-Shield PoC) as previously described^42^. Cells were resuspended in Roswell Park Memorial Institute (RPMI) 1640 (without phenolred, [PAA Laboratories, Inc.])

### NET induction and entrapment assays *A.pp*

Coverslides (12 mm; Thermo Scientific) were coated according to the manufacture’s description with poly-l-lysine (0.001% solution, Sigma Aldrich) for 20 min and washed three times with LPS-free 1× PBS. 250 µl freshly isolated blood-derived porcine neutrophils (5 × 10^5^ cells per well) were seeded in a 24-well plate with slides. As a negative control RPMI only and as a positive control β-methyl-cyclodextrin (Sigma, final concentration 10 mM) was added to the neutrophils in a volume of 250 µl. A similar volume of BALF samples were gently defrosted and sterile filtered (0.4 µm) and also added as stimulus to separate wells. Freshly grown *A.pp* was prepared as described above. *A.pp*-suspension was used to infect neutrophils in separate wells. The plate was centrifuged (370 × *g*, 5 min) and incubated at 37 °C, 5% CO_2_. After 1 and 3 h samples were fixed with 4% paraformaldehyde (final concentration) and NET formation or entrapment was visualized as described below.

### Visualization of NETs, PR-39, and entrapment of bacteria by NETs

NETs were stained as previously described^5^. Briefly, after blocking and permeabilization, neutrophils were incubated with a mouse monoclonal antibody (IgG2a) against DNA/Histone 1 (MAB3864; Millipore 0.55 mg/ml, diluted 1:1000) for 1 h at room temperature. As secondary antibodies a goat anti-mouse antibody (Dye Light488 conjugated highly cross-absorbed, Thermo Scientific; diluted 1:500 in blocking buffer) and goat-anti-rabbit antibody (Alexa633, Thermo Scientific; diluted 1:500 in blocking buffer) were used. To detect PR-39 a polyclonal rabbit anti‐PR‐39 antibody (1:50) was used as previously described^5^. To detect *A.pp* a polyclonal rabbit anti‐*A.pp* St7 antibody (1:50) was used. As secondary antibody goat-anti-rabbit (Alexa633, Thermo Scientific; diluted 1:500 in blocking buffer) was used. Samples were recorded using a Leica TCS SP5 AOBS confocal inverted-base fluorescence microscope with a HCX PL APO ×40 0.75–1.25 oil immersion objective.

### Quantification of NET-releasing cells

For each sample, a minimum of six randomly selected images per independent experiment were acquired and used for quantification of NET-producing cells (NET induction). The cells were counted using ImageJ software (version 1.51q and k, National Institute of Health, USA).

### NETs antimicrobial activity assay

To analyze the antimicrobial activity of NETs against *A.pp*, porcine neutrophils and *A.pp* were co-incubated. *A.pp* was prepared as described above and incubated with 2 × 10^5^ cells in a total volume of 200 µl (48-well plate). As growth control *A.pp* was grown in RPMI alone. Furthermore, a DNase mix with MNe from *S. aureus* (100 mU final, Sigma Aldrich) and/or Deoxyribonuclease I from bovine pancreas (~3000 Units/mg; 100 U/ml or 0.033 mg/ml or 20 U final per well, Sigma Aldrich or ~8000 Units/mg; 100 U/ml or 0.0125 mg/ml or 20 U final per well, SERVA) was added. MNe was used, since SsnA as the main NET-degrading nuclease of *S. suis* is not commercially available. The plate was centrifuged (370 × *g*, 5 min), and incubated at 37 °C, 5% CO_2_. To determine surviving bacteria, samples were mixed, and serious dilutions made at time point 0 min, 1 and 3 h. Dilutions were plated in duplicates on dried boiled-blood agar plates with NAD and incubated over night at 37 °C, 5% CO_2_. Colony forming units (CFU) were counted and the SF was calculated as described in Fig. S2.

To release entrapped bacteria from NETs at the end of experiments, control samples with neutrophils and *A.pp* were treated with 0.5 mU/µl MN (10 min, 37 °C, 5% CO_2_) at the end of incubation (after taking out sample for CFU determination). Efficient degradation of NETs was confirmed by immunofluorescence staining as described above and DNase activity test as described below.

### Intracellular and extracellular staining of *A.pp* and NETs

Coverslides (8 mm; Thermo Scientific) were coated as described above in a 48-well plate. 2 × 10^5^ neutrophils in a total volume of 200 µl were incubated with *A.pp* as described above for 1 and 3 h and afterwards fixed with 4% PFA (final). All staining steps were accomplished at room temperature. Samples were blocked for 20 min in PBS containing 1% BSA and afterwards incubated with polyclonal rabbit anti‐*A.pp* St7 antibody (1:50, 1% BSA PBS) for 1 h. AlexaFluor 633 goat anti‐rabbit (Thermo Scientific; diluted 1:500 in blocking buffer) was used for the following 1 h incubation. The samples were permeabilized for 60 min in PBS containing 0.5% TritonX‐100 and 1% BSA. From this step blocking buffer was used as described^5^. Again, the first antibody was used as described before in combination with DNA/Histone 1 antibody (MAB3864; Millipore 0.55 mg/ml, diluted 1:500) followed by 1 h incubation with Dye Light488 goat anti‐rabbit (Thermo Scientific; diluted 1:500) and Alexa568 (Thermo Scientific; diluted 1:500). Finally, DNA was stained with Hoechst (1:1000; Invitrogen) for 10 min. After staining, the slides were washed and embedded in ProLong Gold antifade without DAPI (Invitrogen). Respective isotype controls were used as described above. Samples were recorded using a Leica TCS SP5 AOBS confocal inverted-base fluorescence microscope with a HCX PL APO lambda blue ×63 1.40 oil immersion UV objective.

### Baclight assay

For live/dead analysis the experiment was conducted as described above in NETs antimicrobial activity assay in 24-well glass bottom plates (MatTek Corporation, Ashland, MA, USA). At the end of incubation cells were stained inside the well with 0.3 µl propidium iodide and 0.3 µl Syto9 (Baclight, Bacterial Viability Kit, Invitrogen) for 15 min in the dark. Microscope settings were adjusted to a dead control with single staining. Randomly 8–12 pictures per sample were taken with Leica TCS SP5 AOBS confocal inverted-base fluorescence microscope with a HCX PL APO lambda blue ×63 1.40 oil immersion UV objective. An uninfected neutrophil sample was included as neutrophil viability control.

### Animal experiment

Pigs have already been experimentally tested in an animal experiment to harvest *A.pp* re-isolates for metabolic fingerprint analysis. Details of the trial were already published for 10 pigs elsewhere including bacteriology of the lungs comparing infected versus uninfected animals^13^. Sixteen 6–9-week-old male castrated pigs (German Landrance) originating from a breeding herd that was routinously tested negative for porcine reproductive and respiratory syndrome virus (PRRSV), *A.pp*, toxigenic *Pasteurella multocida* as well as endoparasite and ectoparasite were used for this study. Pigs were housed according to the guidelines of FELASA and ARRIVE and were randomly assigned to a control (*n* = 8) and infection (*n* = 8) group. Pigs were intramuscularly anaesthetized with 15 mg/kg bodyweight ketamine (Ursotamin, Serumwerk Berneburg AG, Bernburg, Germany) and 2 mg/kg bodyweight azaperon (Stresnil, Janssen-Cilag GmbH, Baar, Switzerland). Pigs were infected as previously described^43^. Briefly, an intratracheal infection was conducted using a fiberoptic bronchoscope under visual control by 5 ml of inoculum containing ~3.0 × 10^6^ CFU *A.pp* per pig (infection group) or with sterile 154 mM sodium chloride as described elsewhere^13^. Clinical scores were recorded at 2 h prior to infection and 2, 5, and 8 h post infection and all pigs were euthanized 8–10 h after infection for necropsy.

Clinical scoring was done and could reach a maximum of 10 points (posture (sitting = 1; lying = 2), coughing (=1), dyspnea (heavy breathing = 1; open-mouth breathing = 2), body temperature (38.0–39.5 °C = 0; >39.5 °C = 1; <38.0 °C = 2), vomiting (=1) and sudden death (=2 extra). One lung lobe was lavaged after separation from the carcass with 100 ml of 154 mM sterile NaCl solution to collect broncho-alveolar lavage fluid (BALF), while the other lung lobe was sampled for histological examination^13^. The BALF was frozen in aliquots at −80 °C. *A.pp*-derived lung changes are not homogeneously spread through the lung as seen in the pictures in Fig. S13. Spots of *A.pp*-infected herds can be detected by eye. For detailed analysis, lung alterations were assessed using a LLS with a possible maximum LLS of 35^44^. Data for the LLS are shown in Table S1.

### Histology staining and immunofluorescence microscopy of lung tissue

Lungs were fixed in formalin and embedded in paraffin for histology and immunofluorescence-based evaluation. Inflammation of lungs was evaluated by haematoxylin and eosin (HE) staining of 2 µm sections. Immunofluorescence staining was conducted for 4 µm sections. Samples were deparaffinized by immersing successively in three changes of xylene for 10 min each and rehydrated by immersing in decreasing concentrations of ethanol (100%, 95%, 70%, each twice for 5 min) and finally two times with water. An antigen retrieval was performed by microwave-heating the slides for 10 min in citrate buffer (0.01 M, pH = 6.0) without boiling. After cooling down for 15 min at room temperature, the slides were washed with 1xTBS three times. Samples were permeabilized 60 min with 0.1% TritonX100 and afterwards washed three times with 1xTBS. The glass was dried and the tissue surrounded with a PapPen. Samples were blocked with a blocking buffer (1% BSA, 5% goat serum, 2% cold water fish gelatine, 0.05% Tween 20, 0.05% Triton X100 in 1xTBS) for 60 min at room temperature. Samples were incubated at room temperature for 1 h with primary antibodies diluted in blocking buffer. Histone–DNA complexes were stained with mouse anti-DNA/Histone1 (Millipore MAB3864, stock 0.55 mg/ml, 1:100 diluted) and rabbit anti-elastase antibody (Abcam, ab1876 stock 10 mg/ml, 1:50 diluted). Respective isotype controls were used to validate background fluorescence intensity. As secondary antibodies a goat anti-mouse antibody (Dye Light488 conjugated highly cross-absorbed, Thermo Scientific; diluted 1:500 in blocking buffer) and goat-anti-rabbit (Alexa633, Thermo Scientific; diluted 1:500 in blocking buffer) were used. After washing, each slide was covered with two drops FluorCare Prolong Gold with DAPI and a cover slide. Histological examinations were recorded with a light microscope. Immunofluorescence samples were recorded using a Leica TCS SP5 AOBS confocal inverted-base fluorescence microscope with a HCX PL APO ×40 0.75–1.25 oil immersion objective. Settings were adjusted with control preparations using an isotype control antibody.

Stained lung slices of three animals per group were analyzed by ImageJ software to determine the percentage of mean fluorescence of red (NE) and green (DNA/Histone) signal based to the blue signal (nuclei). Per animal 10 immunofluorescence microscopy pictures were analyzed.

### LPS detection

BALF was analyzed according to the manufacturer’s recommendation with Pierce LAL Chromogenic Endotoxin Quantitation Kit (ThermoFisher Scientific) to detect lipopolysaccharide.

### Pico green assay

To determine free DNA in BALF, a Pico Green (Invitrogen) assay was conducted according to the manufacturer’s recommendation with the following modifications: BALF samples were tested without centrifugation (non-centrifuged) and after centrifugation (centrifuged, 5 min at 300×*g*) including subsequent harvest of the supernatants of the BALF. A series dilution of calf thymus DNA (1, 0.1, 0.01, 0.001, 0 µg, Sigma Aldrich) was used as standard row. Samples in duplicates were mixed 1:2 in a black flat-bottom 96-well plate with Pico Green (prediluted 1:200 in TE buffer). Afterwards the plate was incubated for 5 min in the dark and then measured in plate reader (TECAN infinite 200pro, Filter 485/535, gain optimized, 25 flashes per well, multiple reads per well, shaking 5 s). The values of DNA were calculated based on the standard row using GraphPad Prism 7.0 software.

### IL-17, PR-39, DNaseI, DNaseII, and lactoferrin ELISA

BALF was analyzed with porcine IL-17 ELISA (Abcam), pig DNase I ELISA (Aviva system Biology Corp.), PR-39 ELISA, pig DNase II ELISA, pig lactoferrin (LF/LTF) ELISA (all MyBioSource Inc.). All tests were used according to the manufacturer’s recommendation.

### DNase activity assay

Visual examination of DNase activity was conducted in all assays after incubation with 1% agarose gel electrophoresis (100 V, 15–30 min) and staining of DNA with RotiSafe (Gelstain ready-to-use, Roth).

DNase activity in BALF samples was tested by 3 h incubation (37 °C) of 50 µl BALF and 0.5 µg calf thymus DNA (Sigma Aldrich). As negative control only PBS was used, as positive control MN from *S. aureus* (Sigma Aldrich) was used.

DNase activity of *A.pp* was tested of freshly grown washed bacteria (prepared as described above) and supernatants of *A.pp*. Bacteria were grown to OD_600nm_ = 0.6 and as overnight culture. 1 µg calf thymus DNA (Sigma Aldrich) was incubated in 70 µl sample (undiluted). Some samples were mixed as indicated 1:2 with 35 µl DNase buffer (pH 7.4, 3 mM CaCl_2_, 3 mM MgCl_2_, 300 mM Tris) to a final reaction volume of 70 µl. All samples were incubated 26 h at 37 °C.

Supernatants from the NET antimicrobial activity test were collected after 3 h and tested for DNase activity. 1 µg calf thymus DNA (Sigma Aldrich) was incubated in 100 µl sample (undiluted) at 37 °C for 21 h or 0.5 µg calf thymus DNA (Sigma Aldrich) was incubated in 50 µl sample (undiluted from NETs antimicrobial activity assay) at 37 °C for 1 h, respectively.

### Determination of bacterial growth in BALF of piglets

To analyze the growth of *A.pp* in sterile filtered BALF (0.2 µm pore size), *A.pp* was freshly grown and washed as described above. The amount of PR-39 in BALF samples in the uninfected was very low, and in the infected group very high (based on ELISA analysis). 500 µl BALF were infected with *A.pp* (1 × 10^7^ CFU/ml) in a 1.5 ml tube and incubated at 37 °C, 5% CO_2_ for 3 h. The CFU was determined by plating on agar plates.

### Bioanalyzer analysis and agarose gel electrophoresis of BALF samples and in vitro samples

DNA fragment size in BALF samples and samples from NETs antimicrobial activity assay were analyzed according to the manufacturer’s recommendation with the high sensitivity DNA kit (Agilent) and bioanalyzer 2100 from Agilent. Furthermore, 25 µl of each sample were load on an 1.3% agarose gel and analyzed with electrophoresis (3 h, 50 V).

### Detection of NAD and adenosine in NETs antimicrobial activity assay

The assay was conducted as described above, including one sample setup without *A.pp*. After 3 h incubation the 48-well plate was centrifuged at 400 × *g* 5 min and the supernatant collected. The pellet was resuspended in 200 µl RPMI and collected. All samples were stored until analysis at −80 °C. Samples were analyzed with NAD ELISA (MyBioSource Inc.) and adenosine assay fluorometric kit (BioVision). All tests were used according to the manufacturer’s recommendation.

### Bacterial strains and growth conditions for co-infection experiments

*Streptococcus (S.) suis* strain 10 is a virulent serotype 2 strain that has been used by different authors for mutagenesis and experimental infections of pigs^45–48^. In addition to the wildtype strain an *in frame* deletion double mutant for the nucleases SsnA and EndASuis was used, named *S. suis* strain 10Δ*endAsuis*Δ*ssnA*^6^, as well as the single mutants *S. suis* strain 10Δ*ssnA*^15^ and *S. suis* strain 10Δ*endAsuis*^6^. Streptococci were grown on Columbia agar plates with 6% sheep blood or in Bacto^TM^ Todd Hewitt broth (THB) (weekly prepared from −80 °C cryo-stock). 10 ml THB medium was inoculated with one colony-forming unit from the Columbia agar plate and incubated for 16 h in a culture tube (12 ml tube, Simport) standing in a box filled with slowly melting crushed ice at 37 °C. On the next day 0.5 ml over-night-culture were transferred to 49.5 ml of pre-warmed THB medium in a 50 ml Falcon tube and afterwards incubated at 37 °C until reaching the OD_600nm_ = 0.6 (mid-log-phase with high production of nucleases). Bacterial cultures were mixed with glycerol (15% final concentration), frozen in liquid nitrogen and stored at −80 °C until usage. The CFU was determined by plating on Columbia agar plates and counting of colonies after 18 h incubation at 37 °C. In the coinfection assay *S. suis* wildtype was used with MOI 1.4–3 and *S. suis* strain 10Δ*endAsuis*Δ*ssnA* with MOI 1.8–3.6.

### NETs antimicrobial activity assay in the presence of a 5′-nucleotidase inhibitor that reduces adenosine production by blocking adenosine synthase

The assay was conducted as described above with *A.pp*. As nuclease the Deoxyribonuclease I from bovine pancreas (20 U final, Serva) was used. In addition samples were co-incubated with adenosine 5′-(α,β-methylene) diphosphate (Sigma Aldrich, final concentration 500 or 250 µM) to reduce adenosine production by blocking adenosine synthase as previously described^24^. Surviving bacteria were determined at time point 0 and 180 min and SF was calculated as described above. Furthermore, the assay was conducted with *H. influenzae* grown and washed as described above. Surviving bacteria were determined at time point 0 and 180 min and SF was calculated as described above.

### *A.pp* growth curve

RPMI medium was supplemented with Isovitale X (BD) and 5% laked horse blood (Fisher Scientific). In a 96 flat-bottom well plate a final volume of 200 µl medium was used. *A.pp* colony material grown on boiled-blood agar plate with NAD was resuspended in 1× PBS and adjusted to an OD_600nm_ = 0.58–0.60. Then, 8 µl out of this suspension was added to each well. One microgram of calf thymus DNA, adenosine 5′-(α,β-methylene) diphosphate (Sigma Aldrich, final concentration 500 µM), MN (500 mU final concentration), or NAD (3 µg final concentration, NAD98-RO ROCHE) was added as indicated. As control PPLO media was used. The plate was incubated in a plate reader with constant temperature (37 °C, TECAN infinite200 pro) and the OD_595nm_ was measured every 30 min after 10 s shaking. As control a blank row was measured and the value was subtracted from each respective sample value. Samples were measured for 19 h and at the end a serious dilution plated on boiled-blood agar plate with NAD (incubated at 37 °C, 5% CO_2_ overnight).

### *A.pp* growth in *S. suis* supernatants

Supernatants of *S. suis* strains were collected after overnight culture and sterile filtered (0.4 µm pore size). *A.pp* was freshly grown and washed as described above. 500 µl supernatants were infected with *A.pp* (1 × 10^7^ CFU/ml) in a 1.5 ml tube and incubated at 37 °C, 5% CO_2_ for 3 h. The CFU was determined by plating on agar plates. Furthermore, the growth was analyzed in a 96-well plate in total volume of 200 µl over time. As media PPLO, THB, and filtered supernatants of *S. suis* strains were used. The plate was incubated in a plate reader with constant temperature (37 °C, TECAN infinite200 pro) and measured as described above. The growth was in addition tested in PPLO, THB, and filtered supernatants of *S. suis* strains (total volume 200 µl with 20 µl of fresh grown and washed *A.pp* as described above in a plate reader with constant temperature and 5% CO_2_ (37 °C, TECAN Spark) for 6.5 h.

### PR-39 killing assay

18 µl washed *A.pp* (final ~1 × 10^7^ CFU/ml) was incubated with 250 µl DNase buffer (Stock 6 mM Mg^2+^, 3 mM Ca^2+^, 300 mM Tris, pH 7.4) and 200 µl PBS. 25 µl DNase (final 100 U/ml) or same amounts of PBS was added as depicted. 7 µl PR-39 (final 3 µM) or water was added as depicted. Samples were incubated 1 or 3 h at 37 °C and 5% CO_2_. The CFU was determined by plating at 0, 1, and 3 h and SF was calculated as described above.

### Dot-blot SsnA

A nitrocellulose membrane (Roth) and two whatman paper (GB002) were pre-wet with Tris-buffered saline Tween 20 (TBST) for 10 min. The wet membrane and paper were placed into a Manifold-I Dot-Blot system (Schleicher & Schuell) following the manufacturer's instructions. 500 µl sample was placed per well under continues vacuum. As controls supernatants of *S. suis* wildtype and ΔssnA were used. BALF of all pigs from the animal experiment as well as from pigs, where BALF samples in the field were taken due to diagnostic reasons were analyzed. 5 min after the last sample was placed into the system, the vacuum was switched off and the membrane was blocked 45 min in 5% non-fat dry milk in TBST. Afterwards a polyclonal rabbit serum raised against rSsnA was used to detect SsnA as described previously^15^ (1% non-fat dry milk in TBST, 1:2000). The membrane was washed four times with TBST. After labeling with a horse-radish peroxidase-conjugated antibody (1% non-fat dry milk in TBST, 1:5000; Cell Signaling Technology), signals were developed using Super Signal West Pico Chemiluminescent Substrate (Thermo Scientific) as recommended by the manufacturer.

#### Whole blood killing assay

Blood were taken from four healthy female 6–7-week-old piglets from the same sow. Per sample 500 µl fresh heparinized blood was filled in a 1.5 ml tube. The blood was infected singly with fresh grown *A.pp* ST2 (2.5 × 10^6^ CFU/ml) or ST7 (3.5 × 10^5^ CFU/ml) as described above. Co-infections were conducted for each *A.pp* strain with *S. suis* wildtype (1.3 × 10^6^ CFU/ml) and *S. suis* ΔssnA (1.3 × 10^6^ CFU/ml), respectively. Tubes were incubated on a rotator (7 rpm, 37 °C, 2 h). At time zero and after 2 h the CFU/ml was determined by plating.

### *H.influenzae*

*H. influenzae* strain CF218 was used in this study. This strain was isolated during routine diagnostics in the Institute of Medical Microbiology, University Hospital Münster, Germany, from the deep throat swab of a patient with cystic fibrosis.

*H. influenzae* was grown on boiled-blood plates with NAD at 37 °C and 5% CO_2_. Liquid culture was grown in fresh supplemented PPLO medium containing 5% blood (Sigma).

For experiments 10 ml supplemented PPLO medium containing 5% blood was inoculated with one colony-forming unit from the boiled-blood plate (weekly new spread out from −80 °C cryostock) and incubated for 17 h in a culture tube shaking at 200 rpm and 37 °C. On the next day 1 ml over-night-culture were transferred to 24 ml of prewarmed supplemented PPLO medium containing 5% blood and afterwards incubated in a rotation shaker with 200 rpm and 37 °C until reaching the OD_600nm_ = 0.55–0.65 (mid-log-phase). Afterwards 2 ml bacteria suspension was centrifuged (2600×*g*, 5 min) and the pellet of bacteria was washed with 1× PBS. The bacteria suspension was adjusted in PBS to an OD_600nm_ = 0.5–0.6. Due to variations occurring based on usage of freshly grown bacteria, an MOI of 0.6–2 was reached for all assays.

### Statistical analysis

Data were analyzed using Excel 2010 and 2016 (Microsoft) and GraphPad Prism 7.0 and 8.1 (GraphPad Software). Normal distribution of data was verified by Kolmogorov–Smirnov normality test (GraphPad software) prior to statistical analysis. Differences between groups were analyzed as described in the figure legends (*P < 0.05, **P < 0.01, ***P < 0.001, ****P < 0.0001).

## Supplementary information

Supplemental Table 4

Supplemental Figure 1

Supplemental Figure 2

Supplemental Figure 3

Supplemental Figure 4

Supplemental Figure 5

Supplemental Figure 6

Supplemental Figure 7

Supplemental Figure 8

Supplemental Figure 9

Supplemental Figure 10

Supplemental Figure 11

Supplemental Figure 12

Supplemental Figure 13

Supplemental Table 1

Supplemental Table 2

Supplemental Table 3
