# Supplementary material for: Degraded neutrophil extracellular traps promote the growth of Actinobacillus pleuropneumoniae
Source: Cell Death Dis. 2019 Sep 10;10(9):657. doi: 10.1038/s41419-019-1895-4 (PMC6736959; doi:10.1038/s41419-019-1895-4)
Supplement: Supplementary file 1 — Supplemental Table 4 [file 41419_2019_1895_MOESM1_ESM.docx]

**Table S4. Clinical case report of *A.pp* infected herd.** A severe clinical case of pleuropneumonia in gilts originating from an *A.pp*-free breeding farm illustrates the impact of *A.pp* and *S. suis* co-infection on the health status of herds.

| Farm details | 1700 sows in a farrow to finishing herd |
| --- | --- |
| History | Periodical losses in fatteners due to *A.pp*. |
| Vaccination | Three weeks prior to farrowing with a farm-specific vaccine containing *A.pp* serotype 2; in addition nursery piglets were vaccinated with the same vaccine in the age of 9 weeks |
| Short-term case history | Gilts were purchased from an *A.pp* negative breeding herd and kept in a quarantine station in approximately 30 km distance from the farrowing herd for 5 weeks. After arrival and three weeks later, gilts were vaccinated twice with a commercial *A.pp* vaccine providing cross protection against all *A.pp* serotypes. Vaccinated gilts were introduced to the sow farm by step-wise adaptation to the unique environment, providing contact with adult sows earliest 4 weeks after end of quarantine. |
| First week | Within this first week of contact to adult sows, a whole group of 20 gilts showed clincal signs of respiratory disease and fever. |
|  | Four sows died. |
|  | Postmortal diagnostic in one sow revealed severe haemorrhagic pneumonia and fibrinous pleuritis. ***A.pp*. serotype 2 as well as *S. suis* serotype 2 were isolated from inflamed lung tissue with high bacterial loads.** |
|  | The veterinarian was forced to treat the whole group of sows with marbofloxacin followed by treatment with doxycycline to avoid further losses. |
| 4 weeks later | The isolated bacterial strains were implemented in a new charge of autologous vaccines. |
| Conclusion | This case clearly demonstrates, that vaccine protection can fail, when specific bacteria-bacteria interactions overcome innate immune reactions with the consequence of fatal disease. |
