# Supplementary material for: Degraded neutrophil extracellular traps promote the growth of Actinobacillus pleuropneumoniae
Source: Cell Death Dis. 2019 Sep 10;10(9):657. doi: 10.1038/s41419-019-1895-4 (PMC6736959; doi:10.1038/s41419-019-1895-4)
Supplement: Supplementary file 2 — Supplemental Figure 1 [file 41419_2019_1895_MOESM2_ESM.docx]

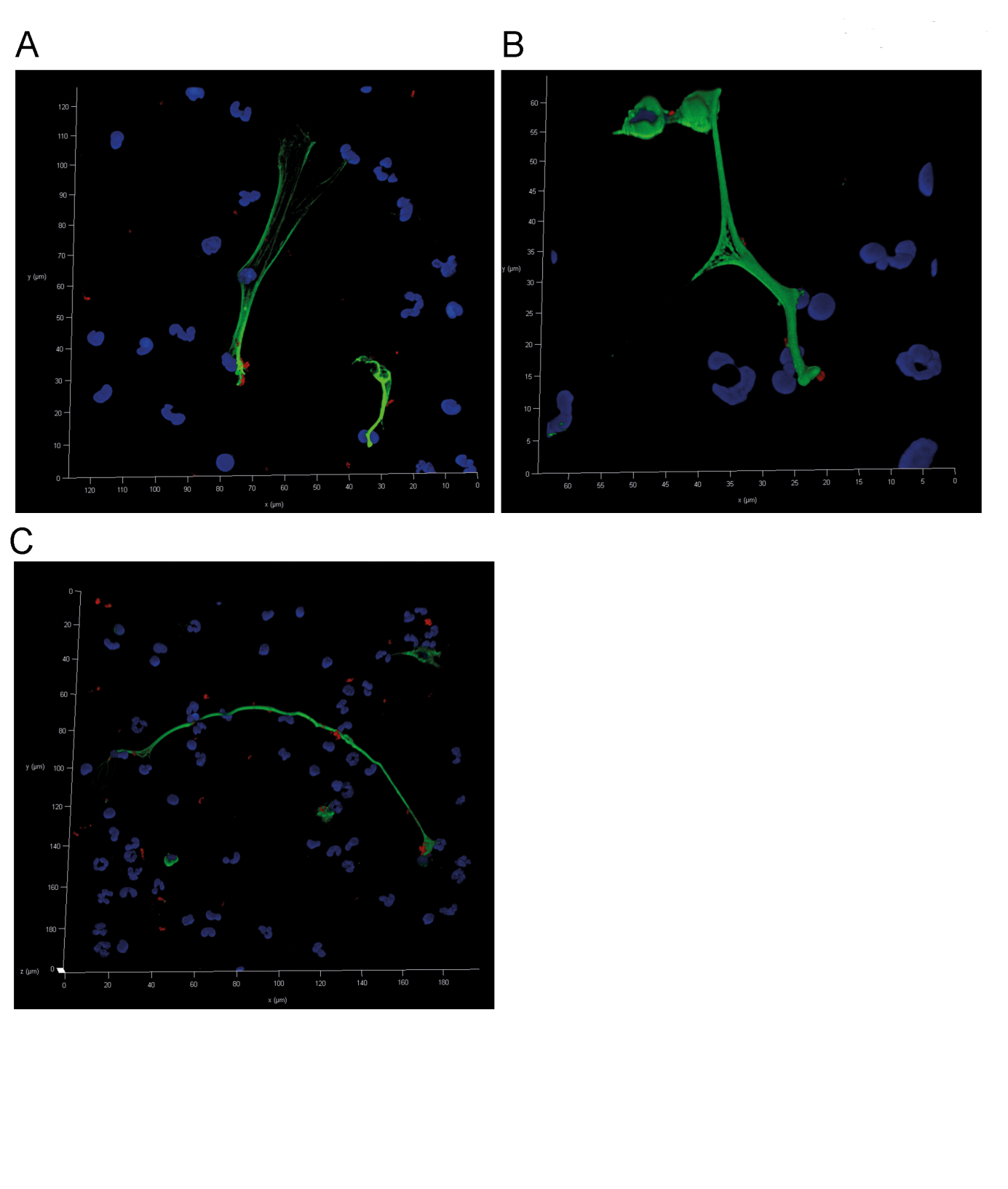


Supplemental figure 1 *A.pp* is partially entrapped in porcine NETs. Primary fresh blood-derived porcine neutrophils were isolated and treated with *A.pp*. After incubation at 37°C and 5 % CO_2_ the cells were fixed. NET and *A.pp* staining for immunofluorescence microscopy was conducted (blue = DNA (Hoechst), green = DNA/histone‐1‐complexes (NETs), red = *A.pp*). For the 3D picture, z-stacks were collected and analyzed with LAS X 3D Version 3.1.0 software from Leica. (A) A total of 37 z-stack pictures (0.13 µm steps) were used and the background set to black by standard software settings. (B) A total of 43 z-stack pictures (0.25 µm steps) were used and the background set to black by standard software settings. (C) A total of 41 z-stack pictures (0.25 µm steps) were used and the background set to black by standard software settings. The isotype control showed no NET or *A.pp* specific signal.
