# Supplementary material for: Degraded neutrophil extracellular traps promote the growth of Actinobacillus pleuropneumoniae
Source: Cell Death Dis. 2019 Sep 10;10(9):657. doi: 10.1038/s41419-019-1895-4 (PMC6736959; doi:10.1038/s41419-019-1895-4)
Supplement: Supplementary file 3 — Supplemental Figure 2 [file 41419_2019_1895_MOESM3_ESM.docx]

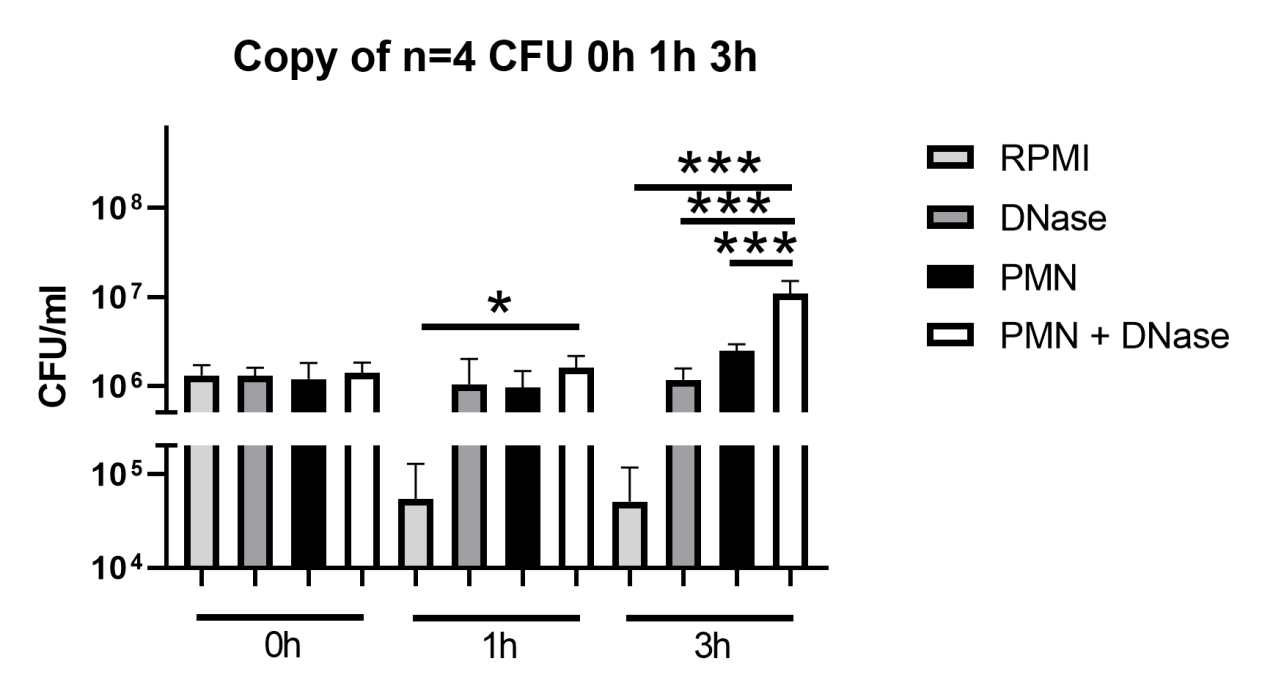


Supplemental figure 2 **Colony forming units from NETs antimicrobial activity assay.** Based on the presented CFU/ml the survival factor (SF) in figure 1 E and F were calculated for 1h (CFU_1h_ / CFU_0h_) and 3h (CFU_3h_ / CFU_0h_). Data shown as mean ± SD (n=4, one-way ANOVA calculation resulted in 0h *P* =* 0.64, 1h *P* =* 0.0124, 3h *P* =* 0.0001. If *P*-value was statistically significant, a Tukey’s multiple comparison test was conducted; (**P* <0.05, ****P* < 0.001,).
