# Supplementary material for: Degraded neutrophil extracellular traps promote the growth of Actinobacillus pleuropneumoniae
Source: Cell Death Dis. 2019 Sep 10;10(9):657. doi: 10.1038/s41419-019-1895-4 (PMC6736959; doi:10.1038/s41419-019-1895-4)
Supplement: Supplementary file 4 — Supplemental Figure 3 [file 41419_2019_1895_MOESM4_ESM.docx]

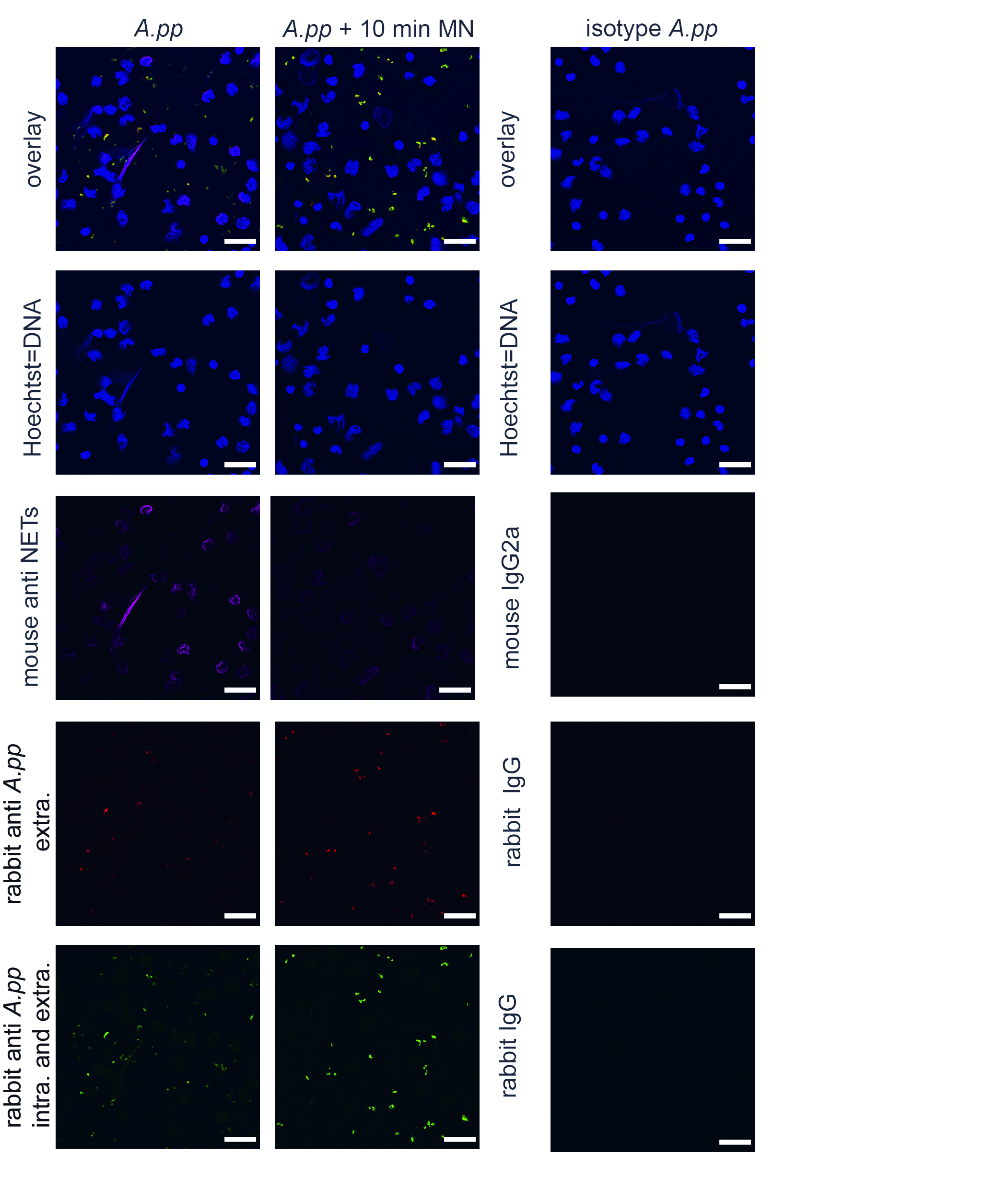


Supplemental figure 3 Single channel pictures for staining of intra- and extracellular *A.pp*. Respective single channels for overlay pictures presented in Fig. 2 (3h) are presented together with the respective isotype control. Red shows extracellular *A.pp*, green shows intracellular *A.pp*, therefore in the overlay extracellular bacteria are yellow, NETs are magenta. MN = micrococcal nuclease, Scale bar = 20 µm.
