# Supplementary material for: Degraded neutrophil extracellular traps promote the growth of Actinobacillus pleuropneumoniae
Source: Cell Death Dis. 2019 Sep 10;10(9):657. doi: 10.1038/s41419-019-1895-4 (PMC6736959; doi:10.1038/s41419-019-1895-4)
Supplement: Supplementary file 5 — Supplemental Figure 4 [file 41419_2019_1895_MOESM5_ESM.docx]

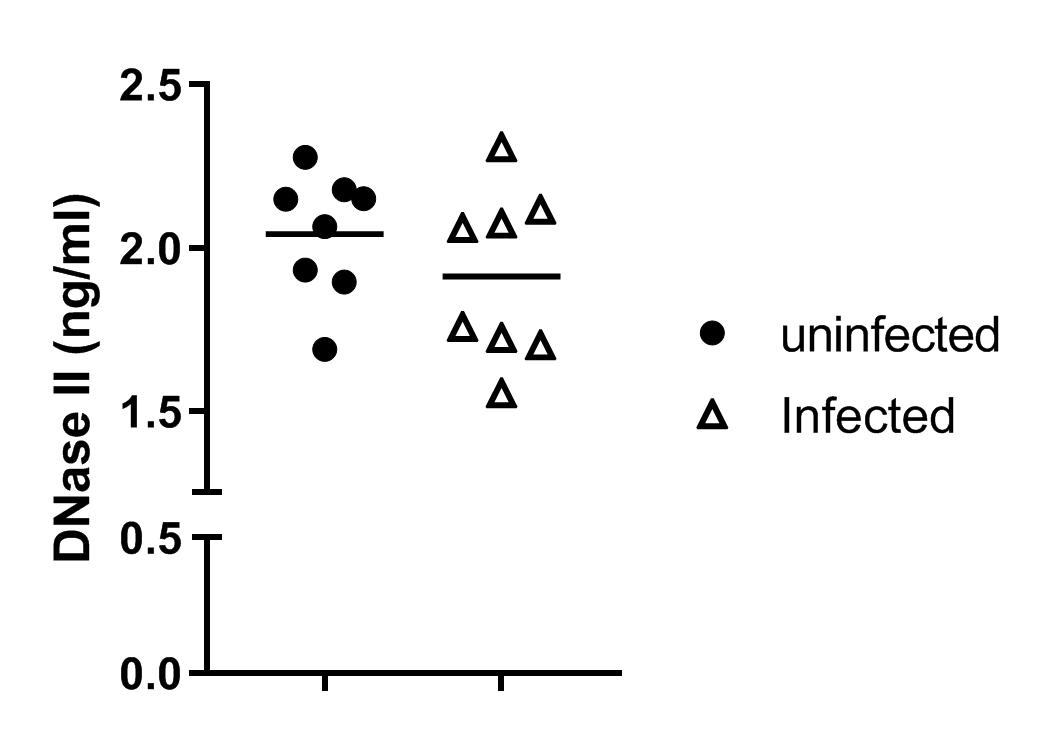


Supplemental figure 4 Porcine DNase II is not increased in BALF of *A.pp*-infected pigs. Porcine DNase II was detected in BALF samples of *A.pp*-infected and uninfected pigs using ELISA. Each sample was measured in duplicate and a mean calculated. Compared results are shown as mean with individual animal values. Data are analyzed with one-tailed unpaired Student‘s t-Test.
