# Supplementary material for: Degraded neutrophil extracellular traps promote the growth of Actinobacillus pleuropneumoniae
Source: Cell Death Dis. 2019 Sep 10;10(9):657. doi: 10.1038/s41419-019-1895-4 (PMC6736959; doi:10.1038/s41419-019-1895-4)
Supplement: Supplementary file 6 — Supplemental Figure 5 [file 41419_2019_1895_MOESM6_ESM.docx]

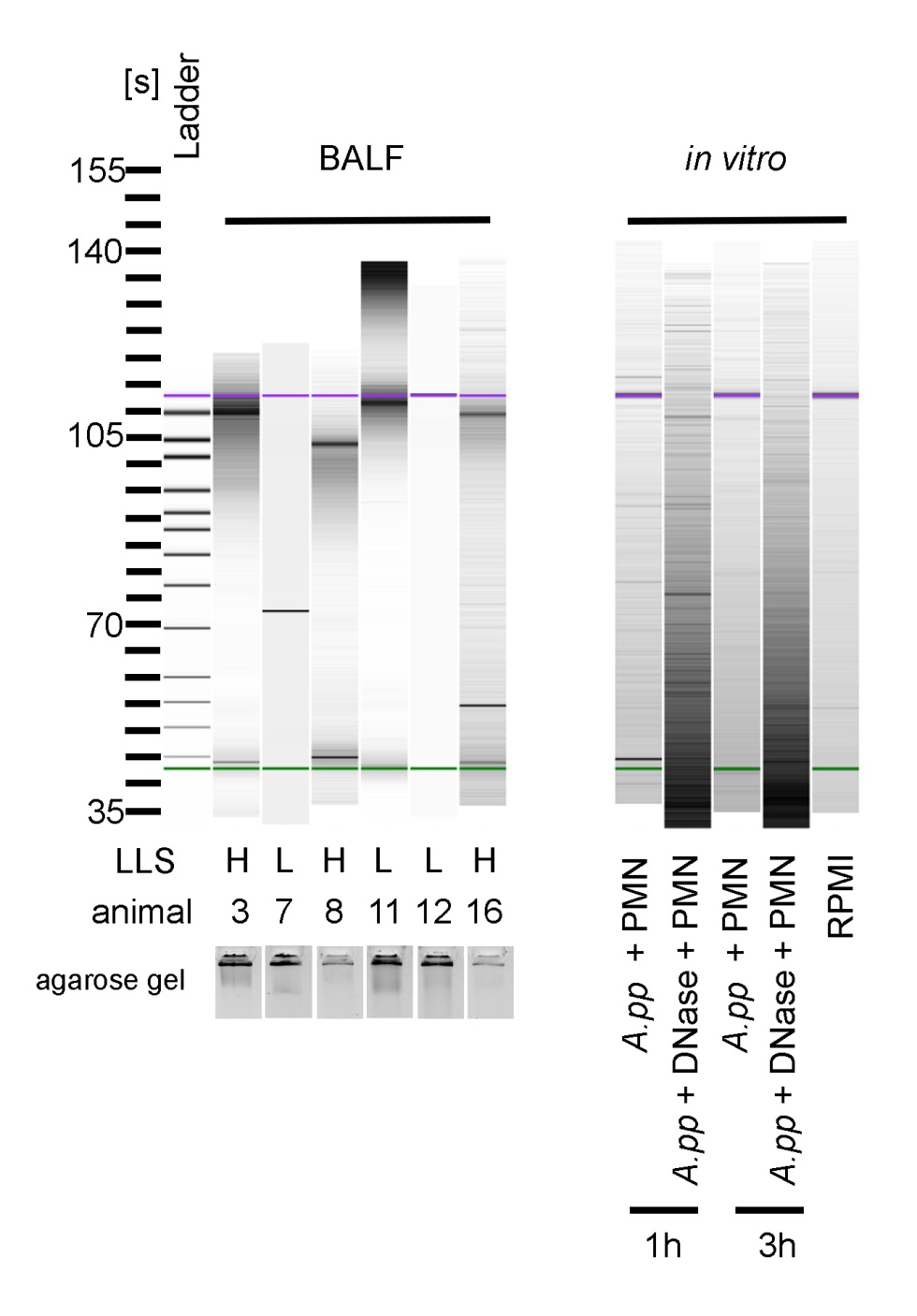


Supplemental figure 5 Bioanalyzer based and gel agarose electrophoresis based analysis of BALF samples and *in vitro* samples. Left panel shows BALF samples of infected animals with high (H) and low (L) lung lesion score (LLS). Animal numbers refer to values presented in supplemental table 1. BALF samples were in addition loaded on an 1.3% agarose gel to identify high molecule DNA fragments.

Right panel shows analysis of collected *in vitro* samples (supernatants from Figure 2A). RPMI was used as negative control. Internal standard is presented as violet (10.380 bp) and green (35 bp) line.
