# Supplementary material for: Degraded neutrophil extracellular traps promote the growth of Actinobacillus pleuropneumoniae
Source: Cell Death Dis. 2019 Sep 10;10(9):657. doi: 10.1038/s41419-019-1895-4 (PMC6736959; doi:10.1038/s41419-019-1895-4)
Supplement: Supplementary file 7 — Supplemental Figure 6 [file 41419_2019_1895_MOESM7_ESM.docx]

**
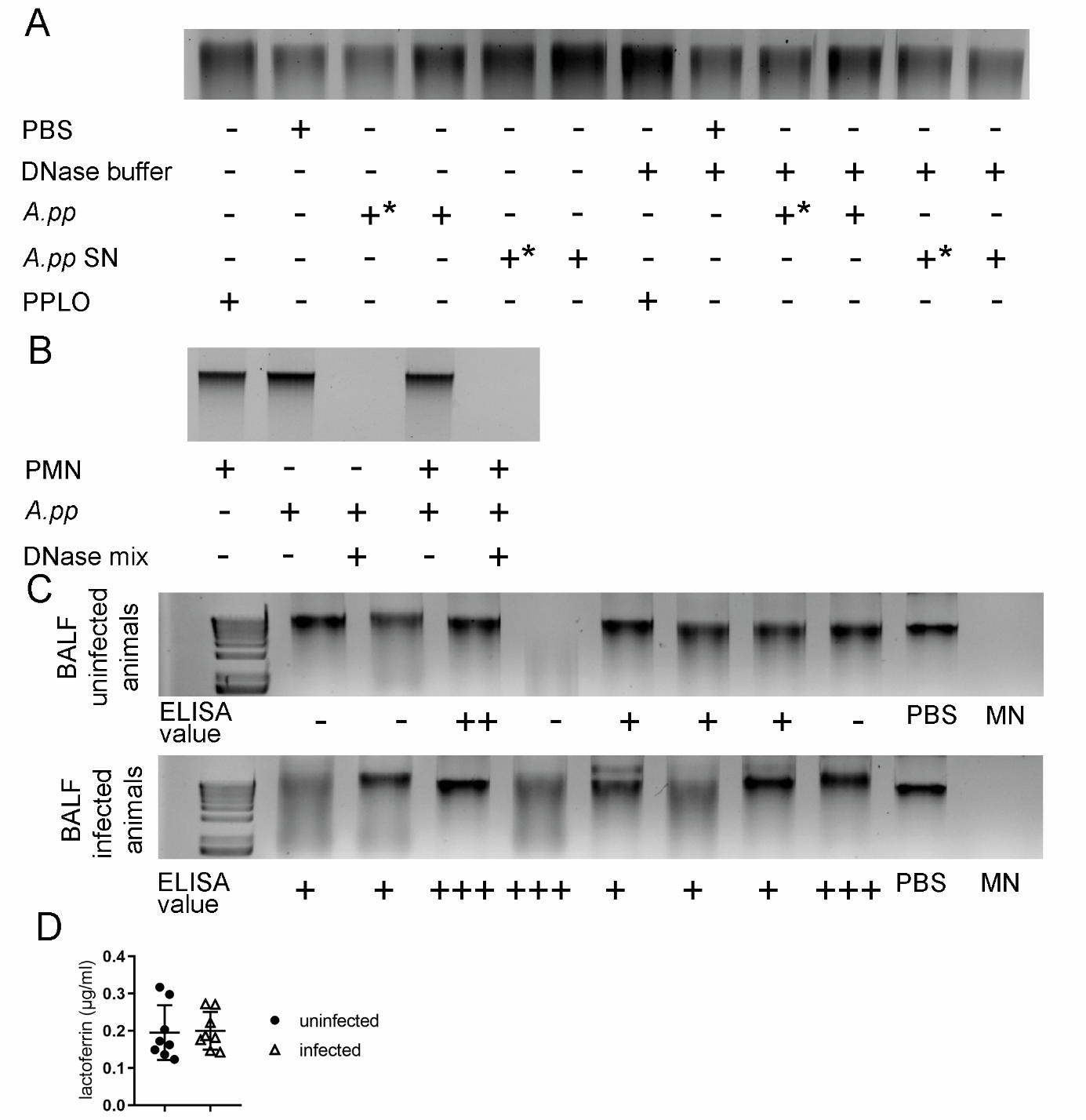
**Supplemental figure 6 **DNase activity of *A.pp* and in BALF.** DNase activity was determined by incubation of calf thymus DNA in samples at 37°C. DNA was visualized with 1% agarose gel electrophoresis. (A) DNase activity of differently grown *A.pp* was determined: *A.pp* = washed *A.pp*, *A.pp* SN = supernatants from *A.pp* grown in PPLO medium, +* = *A.pp* overnight culture, + = *A.pp* samples harvested from midlog phase. DNA was incubated in the samples under different conditions as indicated for 24 h. No *A.pp* dependent DNase activity was detectable. (B) DNase activity of *A.pp* after 3 h stimulation in the NET antimicrobial activity assay (Fig. 1 B and C) was determined in the harvested samples. As a natural stimulation porcine neutrophils (PMN) were used. DNA was incubated in the samples for 21 h. DNase mix was added as positive control in indicated samples. No *A.pp* dependent DNase activity was detectable after natural stimulation. (C) One µg DNA was incubated with BALF of infected and uninfected animals for 3 h. Amount of porcine DNase 1 that was determined by ELISA (Fig. 3C) are presented below the gel (- = < 20 pg/ml, + = < 50 pg/ml, ++ = <100 pg/ml, +++ = > 100pg/ml). PBS was used as negative control, micrococcal nuclease (MN) as positive control. Degradation of DNA in BALF was detectable independent of *A.pp*-infection or presence of pig DNase 1 as measured by ELISA. Therefore DNase of other origin than *A.pp* or host DNase 1 may have been released.

(D) Similar lactoferrin levels are present in BALF of *A.pp* infected animals compared to control animals. BALF was analyzed with an ELISA to determine the amount of lactoferrin (µg/ml). All samples were tested in two independent technical runs. The mean of both runs is shown in the graph. In both groups, BALF of n=8 animals were analyzed. No significant difference was found between both groups (unpaired, one-tailed Student’s t-Test).
