# Supplementary material for: Degraded neutrophil extracellular traps promote the growth of Actinobacillus pleuropneumoniae
Source: Cell Death Dis. 2019 Sep 10;10(9):657. doi: 10.1038/s41419-019-1895-4 (PMC6736959; doi:10.1038/s41419-019-1895-4)
Supplement: Supplementary file 8 — Supplemental Figure 7 [file 41419_2019_1895_MOESM8_ESM.docx]

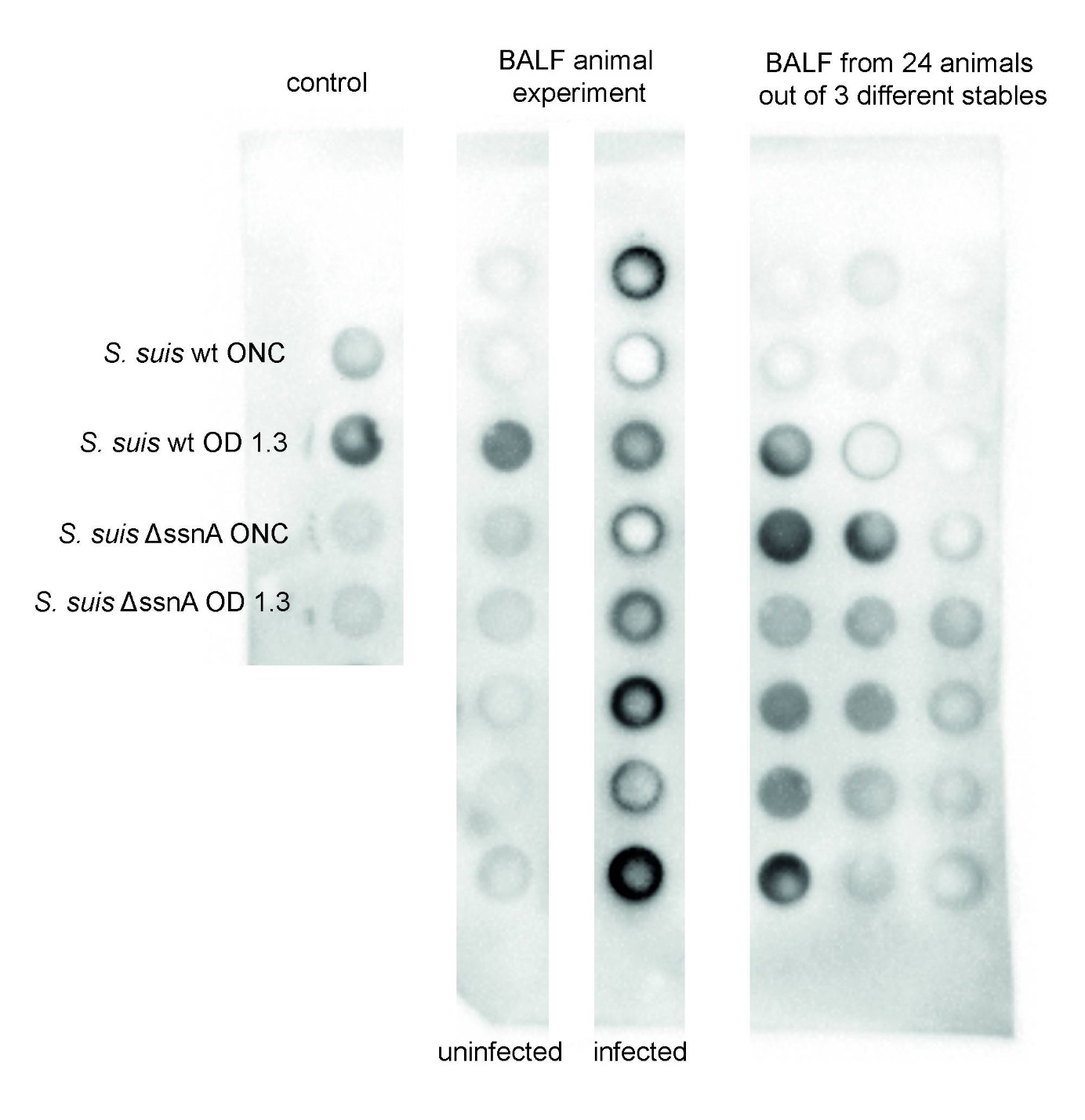
 Supplemental figure 7 *S. suis* nuclease ssnA detection in BALF samples. Supernatants of *S. suis* wildtype (wt) and *S. suis* ΔssnA were collected after overnight culture (ONC) or growth until stationary growth phase (OD 1.3). By dot blot method, samples were put on a nitrocellulose membrane. The used volume was 200 µl *S. suis* samples (left side) and 500 µl each BALF samples (right side). The two first samples of infected BALF samples were not passing complete the membrane. The BALF form the 24 animals (right side) were sampled in clinically healthy pigs on three conventional farms. After blocking the membrane (5% milk powder), a rabbit αssnA was added for 1h at room temperature and detected by HRP conjugated αrabbit antibody (1h, room temperature) and super signal developer. The light grey signal in the *S. suis* ΔssnA is set as negative whereas the dark grey signal in *S. suis* wildtype is set as positive. Therefore, several tested animals show a positive ssnA signal in the BALF samples.
