# Supplementary material for: Degraded neutrophil extracellular traps promote the growth of Actinobacillus pleuropneumoniae
Source: Cell Death Dis. 2019 Sep 10;10(9):657. doi: 10.1038/s41419-019-1895-4 (PMC6736959; doi:10.1038/s41419-019-1895-4)
Supplement: Supplementary file 9 — Supplemental Figure 8 [file 41419_2019_1895_MOESM9_ESM.docx]

**
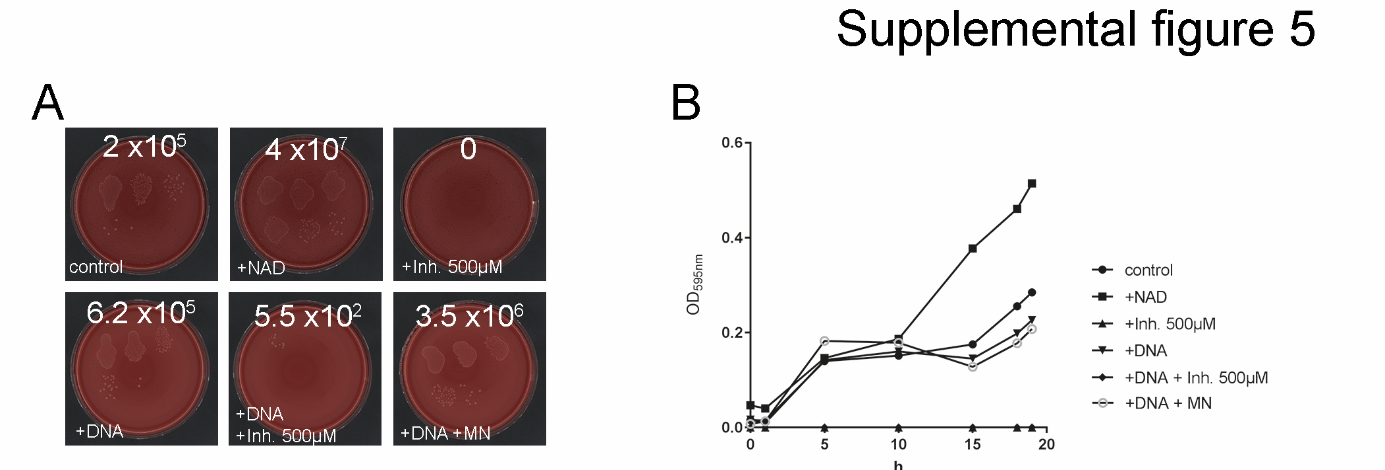
**Supplemental figure 8 **Growth inhibition of *A.pp* by adenosine 5′-(α, β-methylene) diphosphate.** The growth of *A.pp* was tested over time (19 h) in presence of different factors. Control is RPMI supplemented with Isovitale X and 5% laked horse blood. This media was used in all presented samples with the described supplements. The addition of adenosine 5′-(α,β-methylene) diphosphate as an inhibitor of 5´-nucleotidase completely inhibited the growth of *A.pp*. In the presence of DNA and the inhibitor, *A.pp* was slightly growing as shown by counting of colony forming units (5.5 x10^2^ CFU/ml), but no growth was detectable by optical density. (A) After 19 h incubation at 37°C the CFU/ml was determined by plating. Representative agar plates are shown. As growth control *A.pp* was grown in PPLO (2.7x10^8^ CFU/ml, data not shown), whereas is RPMI without any supplementation no growth was detectable (data not shown). (B) Data of growth curves (19 h measurement, data presented only for 0 h, 1 h, 5 h 10 h, 15h, 18 h and 19 h) are presented as mean out of 3 independent experiments (+DNA + MN was only conducted n=2).
