# Supplementary material for: Degraded neutrophil extracellular traps promote the growth of Actinobacillus pleuropneumoniae
Source: Cell Death Dis. 2019 Sep 10;10(9):657. doi: 10.1038/s41419-019-1895-4 (PMC6736959; doi:10.1038/s41419-019-1895-4)
Supplement: Supplementary file 10 — Supplemental Figure 9 [file 41419_2019_1895_MOESM10_ESM.docx]

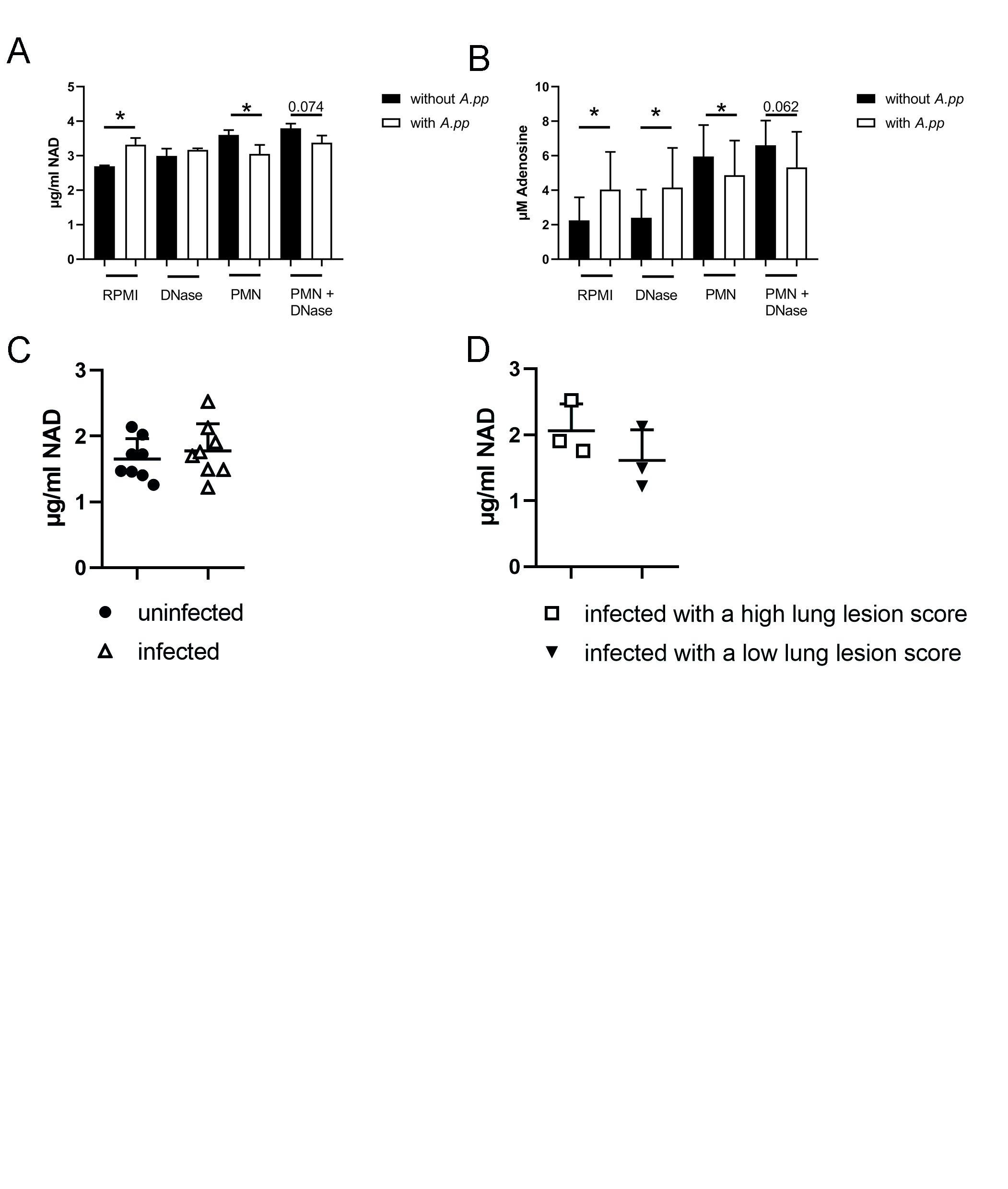
Supplemental figure 9 NAD and adenosine are released by neutrophils and decreased by *A.pp.* NAD was determined with ELISA and adenosine with a colorimetric assay in samples from *A.pp* growth experiments (Fig. 1F). Data presented from n=3 independent experiment and were measured in one technical detection run. Values were determined in experiments with presence of *A.pp*. In A and B the values of incubation with and without *A.pp* (see Fig. 6) are compared for cells plus supernatant (SN). (C) NAD was detected in BALF samples of *A.pp*–infected and uninfected pigs. (D) NAD values are grouped in the infected animals based on the lung lesion score.

Data are presented with mean ± SD and analyzed with one-tailed paired Student‘s t-Test (**P*<0.05) in each treatment group (A and B) or of both groups (C and D). Compared results in C and D are shown with individual animal values.
