# Supplementary material for: Degraded neutrophil extracellular traps promote the growth of Actinobacillus pleuropneumoniae
Source: Cell Death Dis. 2019 Sep 10;10(9):657. doi: 10.1038/s41419-019-1895-4 (PMC6736959; doi:10.1038/s41419-019-1895-4)
Supplement: Supplementary file 11 — Supplemental Figure 10 [file 41419_2019_1895_MOESM11_ESM.docx]

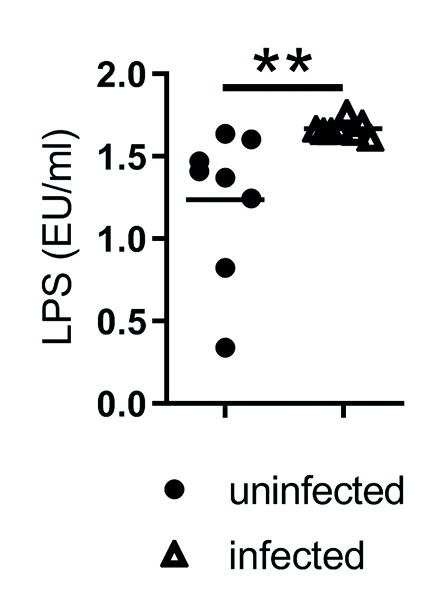


Supplemental figure 10 LPS detection in BALF samples. BALF samples were analyzed using LAL test to quantify level of LPS. Significant higher amount of LPS was detected in BALF of *A.pp*-infected pigs, but in all tested samples LPS was detectable. Compared results of the mean values are presented [**P <0.01, one-tailed unpaired Student‘s t-Test, n=8 each group].
