# Supplementary material for: Degraded neutrophil extracellular traps promote the growth of Actinobacillus pleuropneumoniae
Source: Cell Death Dis. 2019 Sep 10;10(9):657. doi: 10.1038/s41419-019-1895-4 (PMC6736959; doi:10.1038/s41419-019-1895-4)
Supplement: Supplementary file 12 — Supplemental Figure 11 [file 41419_2019_1895_MOESM12_ESM.docx]

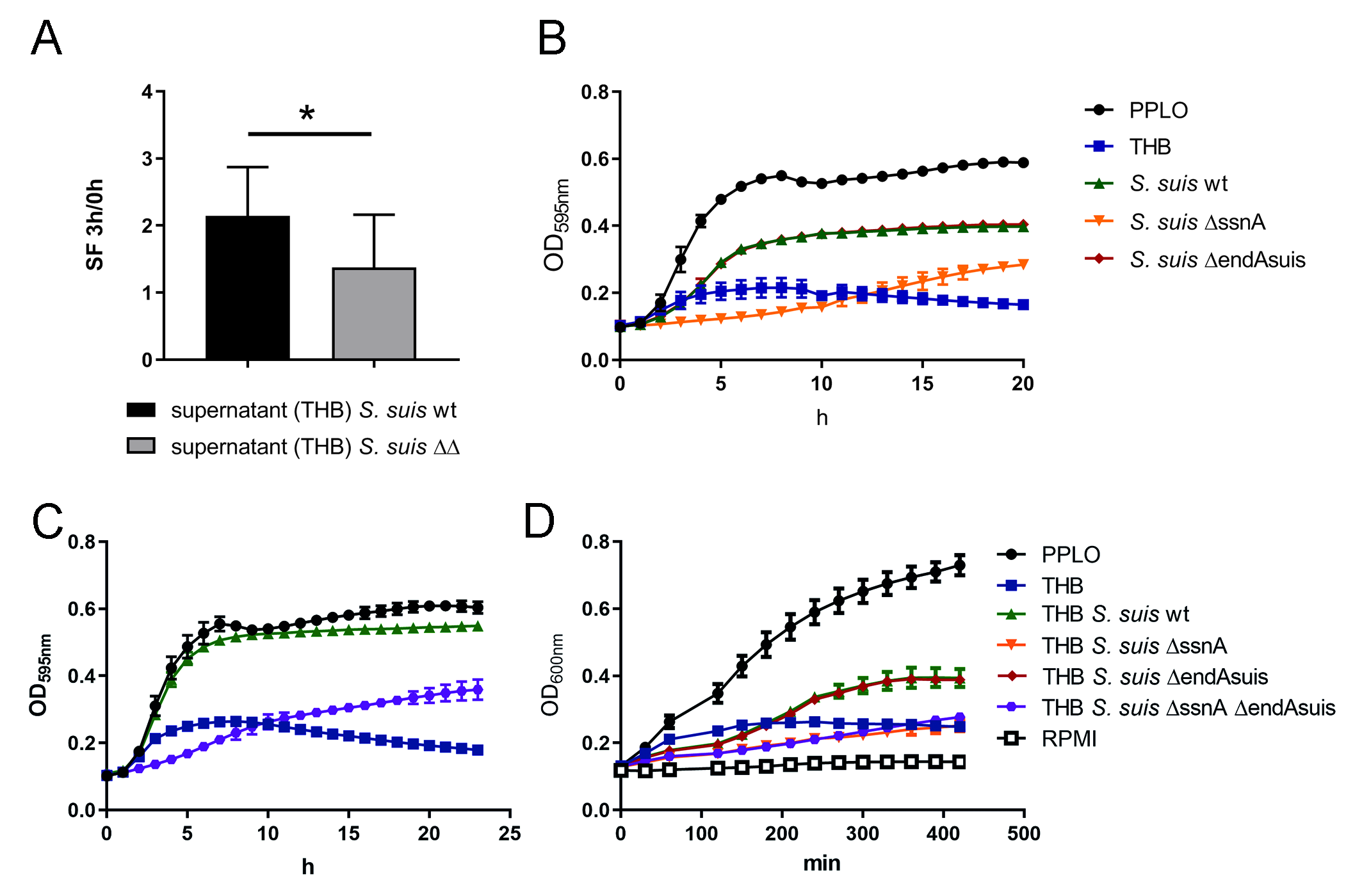
Supplemental figure 11 *A.pp* grows better in presence of *S. suis* nuclease ssnA. Supernatants of *S. suis* wildtype (wt) and *S. suis* nuclease mutants (double mutant ΔssnAΔendAsuis and single mutants ΔssnA or ΔendAsuis) were collected after overnight culture and sterile filtered. (A) *A.pp* was grown in supernatants for 3 h and CFU determined by plating. The survival factor of *A.pp* was calculated based to the CFU at 0h. *A.pp* survives significant better in presence of nuclease ssnA. The assay was conducted in n=3 independent experiments and data are presented as mean ± SD. Statistical analysis was calculated with one-tailed paired Student’s t-test (**P*<0.05). (B) *A.pp* growth was determined by measurement of optical density in a TECAN plate reader. PPLO with isovitale X was used as the standard growth media of *A.pp*. THB (without isovitale X) was used as the standard growth media of *S. suis* and is the background in all supernatants of *S. suis*. Data are presented as mean out of two technical independent runs with triplicate measurement in each run. (C) Data are presented as mean out of one technical run with triplicate measurement. (D) *A.pp* growth was determined by measurement of optical density in a TECAN plate reader with permanent CO_2_. Data are presented as mean out of two technical independent runs with triplicate measurement in each run.
