# Supplementary material for: Degraded neutrophil extracellular traps promote the growth of Actinobacillus pleuropneumoniae
Source: Cell Death Dis. 2019 Sep 10;10(9):657. doi: 10.1038/s41419-019-1895-4 (PMC6736959; doi:10.1038/s41419-019-1895-4)
Supplement: Supplementary file 13 — Supplemental Figure 12 [file 41419_2019_1895_MOESM13_ESM.docx]

**
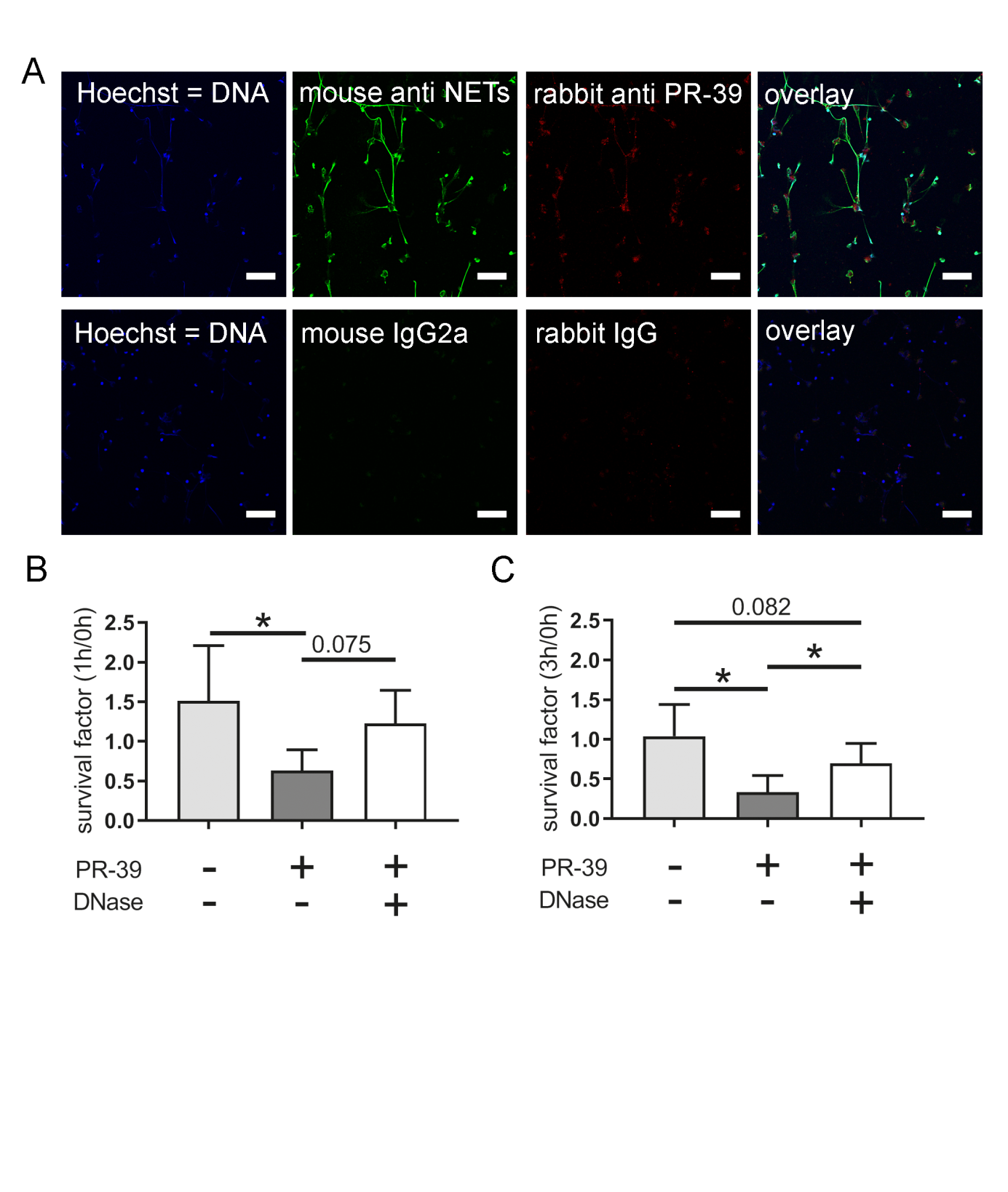
** Supplemental figure 12 **The antimicrobial peptide PR-39 is embedded in porcine NETs released as response to *A.pp* infection** **and PR-39-killing effect of *A.pp* is diminished in presence of DNase.** (A) Primary fresh blood-derived porcine neutrophils were isolated and infected with *A.pp*. After 3h incubation at 37°C and 5% CO_2_ the cells were fixed. Afterwards NET staining for immunofluorescence microscopy was conducted (blue = DNA (Hoechst), green = DNA/histone‐1‐complexes (NETs, red = PR-39). Representative pictures are shown. Staining confirmed a localization of PR-39 in NETs. Respective isotype control staining is depicted in the figure (scale bar = 50µm). (B and C) Washed *A.pp* (1x10^7^ CFU/ml at 0h) was incubated in a total reaction volume of 500µl. The mixture contained DNase buffer (with Mg^2+^, Ca^2+^, pH 7.4) and PBS. Furthermore DNase or PR-39 (final 3µM) was added as depicted or same amounts of water. The CFU was determined by plating at 0h and after 1 h or 3h incubation at 37°C and 5 % CO_2_. Survival factor was calculated as described. PR-39 killed over time *A.pp*. This effect was diminished by adding DNase. This phenomenon can be explained by a usage of DNA from PR-39-killed *A.pp* by growing *A.pp*. The external DNase degrades DNA and 5’ nucleotidase of *A.pp* is active as described. Data of four (B) and three (C) independent experiments are presented.The Statistical analysis was done with one-tailed paired Student‘s t-test (*P<0.05). All values are presented with ±SD.
