# Supplementary material for: Degraded neutrophil extracellular traps promote the growth of Actinobacillus pleuropneumoniae
Source: Cell Death Dis. 2019 Sep 10;10(9):657. doi: 10.1038/s41419-019-1895-4 (PMC6736959; doi:10.1038/s41419-019-1895-4)
Supplement: Supplementary file 14 — Supplemental Figure 13 [file 41419_2019_1895_MOESM14_ESM.docx]

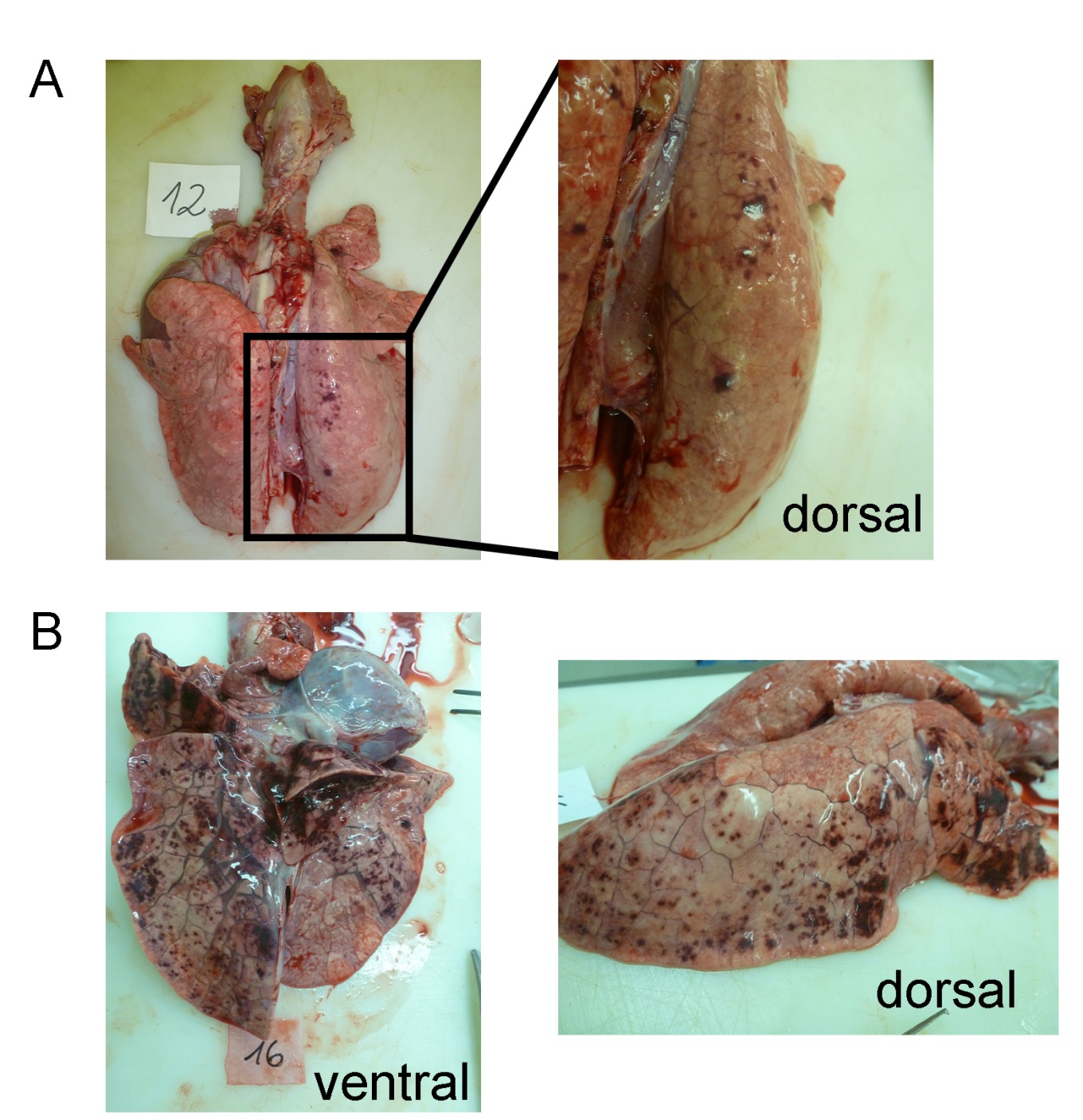


Supplemental figure 13: Macroscopic findings in lungs of *A.pp* infected pigs 8-10 hours after experimental infection. (A) Lung of infected pig with low lung lesion score (3.45). (B) Lung of infected pig with high lung lesion score (18.23). Macroscopic lung alterations were characterized by consolidated red foci reflecting multifocal haemorrhagic lesions accompanied by an interlobular oedema.
