# Supplementary material for: Degraded neutrophil extracellular traps promote the growth of Actinobacillus pleuropneumoniae
Source: Cell Death Dis. 2019 Sep 10;10(9):657. doi: 10.1038/s41419-019-1895-4 (PMC6736959; doi:10.1038/s41419-019-1895-4)
Supplement: Supplementary file 15 — Supplemental Table 1 [file 41419_2019_1895_MOESM15_ESM.docx]

Table S1. Pathological lung lesion score of infected piglets used in this study.

The table presents an overview of the lung lesion score from the 16 analyzed animals^41^. The lung lesion score was determined as described in the methods section.

| **Animal** |  | **lung lesion score** | **infection status** |
| --- | --- | --- | --- |
| 1 |  | 0.00 | non infected |
| 2 |  | 0.00 |  |
| 5 |  | 0.71 |  |
| 6 |  | 0.79 |  |
| 9 |  | 1.42 |  |
| 10 |  | 0.00 |  |
| 13 |  | 0.00 |  |
| 14 |  | 0.00 |  |
|  | **Ø** | **0.37** |  |
| 3 |  | 15.00 | *A.pp* (serotype 2) infected |
| 4 |  | 6.46 |  |
| 7 |  | 3.75 |  |
| 8 |  | 16.01 |  |
| 11 |  | 2.13 |  |
| 12 |  | 3.45 |  |
| 15 |  | 11.03 |  |
| 16 |  | 18.23 |  |
|  | **Ø** | **9.51** |  |
