# Supplementary material for: Degraded neutrophil extracellular traps promote the growth of Actinobacillus pleuropneumoniae
Source: Cell Death Dis. 2019 Sep 10;10(9):657. doi: 10.1038/s41419-019-1895-4 (PMC6736959; doi:10.1038/s41419-019-1895-4)
Supplement: Supplementary file 16 — Supplemental Table 2 [file 41419_2019_1895_MOESM16_ESM.docx]

Table S2. BLAST results of bacterial nucleases in *A.pp*

The table presents an overview of described bacterial nucleases. No of the presented nuclease was detectable in the genome of *A.pp* serotype 2 by NCBI BLAST analysis.

| Bacteria | Nuclease name | Reference |
| --- | --- | --- |
| *Vibrio cholerae* | Xds and Dns | ^1,2^ |
| *Neisseria gonorrhoeae* | Nuc | ^3^ |
| *Staphylococcus aureus* | Nuc | ^4^ |
| *Streptococcus suis* | SsnA | ^5–7^ |
| *Streptococcus pneumoniae* | EndA | ^8–13^ |
| *Streptococcus pyogenes* | Sda1 and MF | ^14–19^ |

**References**

1 Blokesch M, Schoolnik GK. The Extracellular Nuclease Dns and Its Role in Natural Transformation of Vibrio cholerae. *J Bacteriol* 2008; **190**: 7232–7240.

2 Seper A, Hosseinzadeh A, Gorkiewicz G, Lichtenegger S, Roier S, Leitner DR *et al.* Vibrio cholerae Evades Neutrophil Extracellular Traps by the Activity of Two Extracellular Nucleases. *PLoS Pathog* 2013; **9**: e1003614.

3 Juneau RA, Stevens JS, Apicella MA, Criss AK. A Thermonuclease of Neisseria gonorrhoeae Enhances Bacterial Escape From Killing by Neutrophil Extracellular Traps. *J Infect Dis* 2015; **212**: 316–324.

4 Berends ETM, Horswill AR, Haste NM, Monestier M, Nizet V, von Köckritz-Blickwede M. Nuclease expression by Staphylococcus aureus facilitates escape from neutrophil extracellular traps. *J Innate Immun* 2010; **2**: 576–86.

5 Fontaine MC, Perez-Casal J, Willson PJ. Investigation of a Novel DNase of Streptococcus suis Serotype 2. *Infect Immun* 2004; **72**: 774–781.

6 Gómez-Gascón L, Cardoso-Toset F, Amarilla PS, Tarradas C, Carrasco L, Olaya-Abril A *et al.* A new recombinant SsnA protein combined with aluminum hydroxide protects mouse against Streptococcus suis. *Vaccine* 2014; **32**: 6992–6999.

7 de Buhr N, Neumann A, Jerjomiceva N, von Köckritz-Blickwede M, Baums CG. Streptococcus suis DNase SsnA contributes to degradation of neutrophil extracellular traps (NETs) and evasion of NET-mediated antimicrobial activity. *Microbiology* 2014; **160**: 385–395.

8 Lacks S, Greenberg B, Neuberger M. Identification of a Deoxyribonuclease Implicated in Genetic Transformation of Diplococcus pneumoniae. *J Bacteriol* 1975; **123**: 222–232.

9 Beiter K, Wartha F, Albiger B, Normark S, Zychlinsky A, Henriques-Normark B. An Endonuclease Allows Streptococcus pneumoniae to Escape from Neutrophil Extracellular Traps. *Curr Biol* 2006; **16**: 401–407.

10 Midon M, Schafer P, Pingoud A, Ghosh M, Moon AF, Cuneo MJ *et al.* Mutational and biochemical analysis of the DNA-entry nuclease EndA from Streptococcus pneumoniae. *Nucleic Acids Res* 2011; **39**: 623–634.

11 Moon AF, Midon M, Meiss G, Pingoud A, London RE, Pedersen LC. Structural insights into catalytic and substrate binding mechanisms of the strategic EndA nuclease from Streptococcus pneumoniae. *Nucleic Acids Res* 2011; **39**: 2943–2953.

12 Bergé MJ, Kamgoué A, Martin B, Polard P, Campo N, Claverys J-P. Midcell Recruitment of the DNA Uptake and Virulence Nuclease, EndA, for Pneumococcal Transformation. *PLoS Pathog* 2013; **9**: e1003596.

13 Zhu L, Kuang Z, Wilson B a, Lau GW. Competence-independent activity of pneumococcal enda mediates degradation of extracellular DNA and nets and is important for virulence. *PLoS One* 2013; **8**: e70363.

14 Aziz RK, Ismail S a, Park H-W, Kotb M. Post-proteomic identification of a novel phage-encoded streptodornase, Sda1, in invasive M1T1 Streptococcus pyogenes. *Mol Microbiol* 2004; **54**: 184–197.

15 Walker MJ, Hollands A, Sanderson-Smith ML, Cole JN, Kirk JK, Henningham A *et al.* DNase Sda1 provides selection pressure for a switch to invasive group A streptococcal infection. *Nat Med* 2007; **13**: 981–985.

16 Buchanan JT, Simpson AJ, Aziz RK, Liu GY, Kristian S a, Kotb M *et al.* DNase Expression Allows the Pathogen Group A Streptococcus to Escape Killing in Neutrophil Extracellular Traps. *Curr Biol* 2006; **16**: 396–400.

17 Chang Y-C, Uchiyama S, Varki A, Nizet V. Leukocyte Inflammatory Responses Provoked by Pneumococcal Sialidase. *MBio* 2012; **3**: e00220-11-e00220-11.

18 Iwasaki M, Igarashi H, Yutsudo T. Mitogenic factor secreted by Streptococcus pyogenes is a heat-stable nuclease requiring His122 for activity. *Microbiology* 1997; **143**: 2449–2455.

19 Sriskandan S, Unnikrishnan M, Krausz T, Cohen J. Mitogenic factor ( MF ) is the major DNase of serotype M89 Streptococcus pyogenes. 2000; : 2785–2792.
