# Supplementary material for: Degraded neutrophil extracellular traps promote the growth of Actinobacillus pleuropneumoniae
Source: Cell Death Dis. 2019 Sep 10;10(9):657. doi: 10.1038/s41419-019-1895-4 (PMC6736959; doi:10.1038/s41419-019-1895-4)
Supplement: Supplementary file 17 — Supplemental Table 3 [file 41419_2019_1895_MOESM17_ESM.docx]

**Table S3: Prevalence of lung co-infections with *S. suis* and *A.pp* in pigs with respiratory disorders**

| *A.pp* serotype | Lungs positive for  *A.pp* | | Lungs positive for *S. suis* and *A.pp* | | Lungs positive for *S. suis* | |
| --- | --- | --- | --- | --- | --- | --- |
|  | n | % | n | % | n | % |
|  | **223** | **40** | **61** | **11%** | **275** | **49** |
| Not typeable | 53 | 23.8 | 15 | 24.6% |  |  |
| 1 | 1 | 0.4 | 1 | 1.6 |  |  |
| 2 | 111 | 49.8 | 33 | 54.1 |  |  |
| 5 | 10 | 4.5 |  |  |  |  |
| 6 | 2 | 0.9 | 1 | 1.6 |  |  |
| 7 | 1 | 0.4 |  |  |  |  |
| 9 | 21 | 9.4 | 5 | 8.2 |  |  |
| 12 | 2 | 0.9 | 2 | 3.3 |  |  |
| 8/15 | 20 | 9.0 | 3 | 4.9 |  |  |
| multiple | 2 | 0.9 | 1 | 1.6 |  |  |

The table summarizes data from diseased swine sent for routine diagnostics to the Field Station for Epidemiology, University of Veterinary Medicine Hannover from autumn 2016 until the end of 2018. Pigs originated from farms in the swine dense region in the north-western part of Germany. All findings and diagnostic results are routinely registered within a laboratory information system (LabControl, Version 2002, Ticono-Software, Hannover, Germany). Retrospectively, data were filtered for positive microbiological findings of *A.pp*. and / or *S. suis* in the lung. The resulting data set was exported to Excel, Version 2010 (Microsoft Corporation, Albuquerque, USA) for evaluation of the proportion of mono-and coinfections.

In a total of 559 pigs approximately 11% showed co-infection of *A.pp* and *S. suis* in the lung. *S. suis* isolates from lung tissue were not further characterized. Distribution of *A.pp* serotypes were similar in pigs co-infected with *S. suis* and pigs only positive for *A.pp*.
